# STRICTA: is it time to do more?

**DOI:** 10.1186/s12906-015-0714-4

**Published:** 2015-06-20

**Authors:** Lizhou Liu, Margot Skinner, Suzanne M McDonough, Priya Kannan, George David Baxter

**Affiliations:** Centre for Health, Activity and Rehabilitation Research, School of Physiotherapy, University of Otago, PO Box 56, 325 Great King Street, Dunedin, 9054 New Zealand; Centre for Health and Rehabilitation Technologies, Institute of Nursing and Health Research, School of Health Sciences, University of Ulster, Shore Road, Newtownabbey, Co Antrim BT37 0QB UK

**Keywords:** STRICTA, Acupuncture, Reporting quality, Reporting guidelines, Implementation

## Abstract

**Background:**

In order to facilitate the completeness and transparency of reporting on randomized controlled trials undertaken using acupuncture interventions, a consensus group of international experts developed the *Standards for Reporting Interventions in Controlled Trials of Acupuncture* (STRICTA) in 2002. This reporting guideline was updated in 2010, and was applicable to a broader range of acupuncture research, including uncontrolled trials and case reports. Subsequent evaluations have noted limitations on the impact of STRICTA in the reporting quality of acupuncture trials, and the description of acupuncture details remains poor. Thus improvement in the efficacy of the STRICTA guidelines is called for.

**Discussion:**

We explored the STRICTA guidelines from four aspects, including the development procedure, validity assessment, endorsement and adherence, and citation situation. Based upon these findings, we provided five potential suggestions for further development of STRICTA.

**Summary:**

STRICTA are valid reporting guidelines based on robust methodology and scientific content. However specific implementation strategies including: updating the STRICTA checklist; improving the STRICTA reporting efficiency; consistency with implementing the “Instructions for authors” for journals; establishing global STRICTA research centers; and expanding the STRICTA website, are needed. Such strategies will improve their utilization and impact positively on the quality of reporting on acupuncture research outcomes.

**Electronic supplementary material:**

The online version of this article (doi:10.1186/s12906-015-0714-4) contains supplementary material, which is available to authorized users.

## Background

In 2001, an international group of acupuncture researchers developed a recommendation entitled *Standards for Reporting Interventions in Controlled Trials of Acupuncture* (STRICTA) to address reporting issues of controlled trials using acupuncture. It was expected that the use of STRICTA would bring better quality reporting on acupuncture interventions, help the interpretation and analysis, and enable research replications.

Seven years after the initial introduction of STRICTA, Prady *et al* explored the possible impact of STRICTA on the reporting of acupuncture interventions. They conducted systematic reviews with before-and-after approaches to examine whether more STRICTA items were fulfilled in randomized controlled trials (RCTs) over time. Their findings showed that the publication of STRICTA did not have the anticipated impact in that the description of acupuncture trials and the reporting quality remained poor [1]. Four years later, Hammerschlag *et al* developed a comprehensive reporting quality assessment tool (Oregon CONSORT STRICTA Instrument) to evaluate the quality of reporting of RCTs on the use of acupuncture. It was found that although the STRICTA-based reporting improved by 17 % over the period from 1997 to 2007, there was a continual need to enhance the quality of reporting details specific to acupuncture intervention [2]. In the past three years, there has been an increasing tendency to assess the potential effects of STRICTA on the reporting of acupuncture for various clinical diseases, including the diabetic peripheral neuropathy, cancer pain, and neck disorders. However, similar conclusions have been drawn. It would appear that STRICTA have failed to bring the reporting of acupuncture interventions to an acceptable level, and specific implementations are needed to improve the efficacy of STRICTA [3–5].

In order to investigate a potential way forward to improve on the reporting, we considered the STRICTA guidelines from four different aspects (development procedure, validity assessment, endorsement and adherence, and citation situation), and have made recommendations for potential approaches for further development.

## Discussion

### Development of STRICTA

In July 2001, a consensus group of international experts worked on the development of a standard for reporting on RCTs undertaken using acupuncture interventions. The experts comprised academic researchers and experienced acupuncturists, as well as editors of five key Complementary and Alternative Medicine (CAM) journals (*Acupuncture in Medicine, Clinical Acupuncture and Oriental Medicine, Complementary Therapies in Medicine*, *Journal of Alternative and Complementary Medicine*, and *Medical Acupuncture*). The STRICTA guidelines were subsequently published in late 2001 and early 2002 [6–10]. Acting as an extension of the *Consolidated Standards of Reporting Trials* (CONSORT) statement [11], STRICTA were divided into six categories with 19 sub-items to address the specific needs of adequately describing acupuncture interventions: acupuncture rationale, needling details, treatment regimen, co-interventions, practitioner background, and control interventions. It was anticipated by the development team that the full reporting of these items would enhance accurate analysis and interpretation, and ease replication of acupuncture research. To broaden their use internationally the standards were subsequently translated into Chinese, Japanese, and Korean.

As with any such standard, the STRICTA need timely, ongoing review and updating. Seven years after the original release of STRICTA, two reviews [1, 12] were published. These assessed the utility and impact of the STRICTA guidelines on the quality of reporting for acupuncture trials. Both reviews recommended STRICTA be revised to reduce the ambiguity of items. The revision process was initiated in 2008 and involved a collaboration of the STRICTA Group, the CONSORT Group, the Chinese Cochrane Centre, and a range of professionals with specific expertise, including acupuncturists, physicians, academic personnel, and journal editorial board members [13].

Through a multi-staged procedure of expert consultation, consensus workshops, draft editing, and finalization, the revised STRICTA guidelines were agreed upon, and the final document was co-published in 2010 by six medical journals (*Acupuncture in Medicine*, *Australian Journal of Acupuncture and Chinese Medicine*, *Journal of Alternative and Complementary Medicine*, *Journal of Evidence-Based Medicine*, *Medical Acupuncture*, and *PLoS Medicine*) [14–19]. As a standalone checklist STRICTA 2010 became an official extension to CONSORT containing six categories with 17 sub-items. In order to minimize misunderstanding and ambiguity, specific explanations were added for each sub-item, and examples of ‘good reporting’ were included. The updated STRICTA were applicable to a broad range of clinical research, including RCTs (same as STRICTA 2002), uncontrolled studies, and case reports. Furthermore the standards were also translated into a fourth language, Russian.

### Validity of STRICTA

Any reporting guidelines should meet minimum standards for validity including a robust methodology and scientifically validated content. A large number of reporting guidelines have been developed during the past decade [20]; however, the quality of these is not always clear: one systematic review evaluated 81 reporting guidelines, and noted problems with development, based upon questionable methodologies [21]. The development approach has not been independently analyzed for STRICTA, as a reporting guideline. To address this issue, we used two frameworks suggested by the Enhancing the Quality and Transparency of Health Research (EQUATOR) network (http://www.equator-network.org) [22]: 1) *Guidance for Developers of Health Research Reporting Guidelines* [21] which provides prospective reporting guideline developers with widely accepted methodologies during the development procedure, and 2) the *Template for Intervention Description and Replication (TIDieR)* [23], which recommends the minimal essential items for describing an intervention. It was hypothesized that with the help of the two frameworks we could provide a quantitative basis for assessment of the STRICTA 2010 based on the robustness of the guideline development process as well as the content integrity.

The STRICTA guidelines 2010 [14] were used as the primary assessment material; in addition, the reference list and the official website of STRICTA (http://www.stricta.info) [24] were scanned to identify further articles and/or information concerning its development process. As it was an initial evaluation, we did not contact the authors to determine whether missing items were completed and were available but not reported. We converted the two frameworks [21, 23] into two validity assessment checklists. Items were closely aligned with the original recommendations, and the answers could be simply given as “Yes” or “No”. Prior to assessment, each topic on the checklists was intensively discussed to achieve consensus on interpretation; two reviewers (LL and PK) then independently rated the validity of the STRICTA. The inter-rater reliability was calculated using the kappa statistic; disagreements were resolved by discussion, and an independent decision was obtained from a third author (GDB) when disagreement persisted.

Four relevant papers [1, 12–14] plus the STRICTA website were identified and served as primary source for assessment. Agreement of the reviewers for the two checklists was regarded as excellent for independent reviews, with a kappa index of 0.785 for the development process and 0.873 for the content integrity. After discussion the reviewers reached consensus giving a kappa index of 1. Table 1 and Table 2 present the assessment results. For the two checklists, the STRICTA guidelines (2010) satisfied over half of the items (11/18 for the guideline development process, and 8/12 for the content integrity). While the STRICTA group publicized details regarding the development procedures for the initial steps, the post-meeting and the post-publication activities, details on the transparency of the pre-meeting activities and the face-to-face consensus meeting were mostly not disclosed. As for the content validity of the STRICTA 2010, four missing items revealed room for improvement, namely Item 6: delivery modes description; Item 10: intervention modifications explanation; Item 11: planned intervention fidelity assessment; and Item 12: actual results.

**Table 1:** Validity assessment of the STRICTA 2010 on reporting guidelines development process^※^

Step	Item No.	Item	Satisfaction of STRICTA 2010
Initial steps	1	Identify the need for a guideline	Yes
	2	Review the literature	Yes
	3	Obtain funding for the guideline initiative	Yes
Pre-meeting activities	4	Identify participants	Yes
	5	Conduct a Delphi exercise	No
	6	Generate a list of items for consideration at the face-to-face meeting	No
	7	Prepare for the face-to-face meeting	No
Face-to-face consensus meeting itself	8	Present and discuss results of pre-meeting activities and relevant evidence	No
Post-meeting activities	9	Develop the guidance statement	Yes
	10	Develop an explanatory document (E&E)	Yes
	11	Develop a publication strategy	Yes
Post-publication activities	12	Seek and deal with feedback and criticism	Yes
	13	Encourage guideline endorsement	Yes
	14	Support adherence to the guideline	No
	15	Evaluate the impact of the reporting guidance	No
	16	Develop Web site	Yes
	17	Translate guideline	Yes
	18	Update guideline	No

^※^Structured according to the EQUATOR *Guidance for Developers of Health Research Reporting Guidelines* [21]

E&E: explanation and elaboration

**Table 2:** Validity assessment of the STRICTA 2010 on content integrity*

Item No.	Item	Satisfaction of STRICTA 2010
1	Brief name	Yes
	Provide the name or a phrase that describes the intervention	
2	Why	Yes
	Describe any rationale, theory, or goal of the elements essential to the intervention	
3	What (materials)	Yes
	Describe any physical or informational materials used in the intervention, including those provided to participants or used in intervention delivery or in training of intervention providers. Provide information on where the materials can be accessed (such as online appendix, URL)	
4	What (procedures)	Yes
	Describe each of the procedures, activities, and/or processes used in the intervention, including any enabling or support activities	
5	Who provided	Yes
	For each category of intervention provider (e.g. psychologist, nursing assistant), describe their expertise, background and any specific training given	
6	How	No
	Describe the modes of delivery (e.g. face-to-face or by some other mechanism, such as internet or telephone) of the intervention and whether it was provided individually or in a group	
7	Where	Yes
	Describe the type(s) of location(s) where the intervention occurred, including any necessary infrastructure or relevant features	
8	When and how much	Yes
	Describe the number of times the intervention was delivered and over what period of time including the number of sessions, their schedule, and their duration, intensity or dose	
9	Tailoring	Yes
	If the intervention was planned to be personalised, titrated or adapted, then describe what, why, when, and how	
10^∮^	Modifications	No
	If the intervention was modified during the course of the study, describe the changes (what, why, when, and how)	
11	How well (planned)	No
	If intervention adherence or fidelity was assessed, describe how and by whom, and if any strategies were used to maintain or improve fidelity, describe them	
12^∮^	How well (actual)	No
	If intervention adherence or fidelity was assessed, describe the extent to which the intervention was delivered as planned	

^*^Structured according to the *Template for Intervention Description and Replication (TIDieR)* [23]

^∮^Not applicable to a protocol

### Endorsement and adherence to STRICTA

Currently nine international medical journals endorse the STRICTA (2010), including seven CAM specialties and two general medicine journals. A full list of participating journals and their STRICTA publications are available at http://www.stricta.info/journals.html [25].

In order to investigate adherence to STRICTA, we scanned “*Instructions to Authors*” on the websites of those journals that endorse the standard, and extracted relevant information. In addition, in order to examine the awareness of reporting guidelines of those journals, where there was mention of other research reporting guidelines and/or the EQUATOR network this was also extracted. Of the nine journals, six mentioned STRICTA in the online version of “*Instructions to Authors*” (Table 3). However, the majority of journals (n = 5) did not make it an explicit requirement to use STRICTA for potential manuscripts. Only one journal, *Medical Acupuncture*, enforced strict use of the checklist as a compulsory requirement for article submission. It elaborated on the need to adhere to STRICTA throughout the process of manuscript preparation, including appropriate sequencing of the STRICTA items to follow, and the six STRICTA categories required in the methodology section. In regard to referencing, the majority of journals (n = 5) referred to the current STRICTA website, but one referred to a URL currently inaccessible. Two journals included the full STRICTA (2010) Guidelines (one of which was via the STRICTA website). The EQUATOR network was referenced by only two of the endorsing journals, and other relevant reporting guidelines (e.g. CONSORT, PRISMA, and STARD) were also recommended for use by six journals.

**Table 3:** “Instructions to authors” of journals that endorsed STRICTA

Journal	Mention of STRICTA	Specific requirement of STRICTA (quote)	STRICTA website link	Appropriate reference	Mention of EQUATOR	Other reporting guidelines
Acupuncture in Medicine	Yes	*All acupuncture treatment should be described according to the current STRICTA recommendations*	No, but hyperlink to the STRICTA checklist	Yes	Yes	CONSORT, PRISMA, MOOSE, STROBE, STARD, SQUIRE
Australian Journal of Acupuncture and Chinese Medicine	Yes	*The reporting of acupuncture treatment needs to follow STRICTA guidelines*	Yes, but inaccessible URL	NR	No	CONSORT, QUOROM
Deutsche Zeitschrift für Akupunktur/German Journal of Acupuncture and Related Techniques	Yes	*For better reporting of acupuncture trial interventions, please adhere to STRICTA*	Yes	NR	No	No
Journal of Alternative and Complementary Medicine	Yes	*For controlled trials of acupuncture, use our preferred reporting criteria based on the STRICTA guidelines*	Yes	No (Inappropriate Title)	No	CONSORT
Journal of Integrative Medicine	No	NR	No	NR	No	CONSORT, PRISMA
Journal of Evidence-Based Medicine	Yes	*All acupuncture treatment should be described according to the current STRICTA recommendations*	Yes	Yes	No	CONSORT, PRISMA
Korean Journal of Acupuncture	No	NR	No	NR	No	No
Medical Acupuncture	Yes	*All papers must adhere to the new STRICTA 2010 requirements*	Yes	NR	No	No
PLoS Medicine	No	NR	No	NR	Yes	CONSORT, PRISMA, STARD, STROBE, MIAME, MIBBI, ARRIVE

NA: not reported

STRICTA: Standards for Reporting Interventions in Clinical Trials of Acupuncture; EQUATOR: Enhancing the Quality and Transparency of Health Research; CONSORT: Consolidated Standards of Reporting Trials; PRISMA: Preferred Reporting Items for Systematic Reviews and Meta-Analyses; MOOSE: Meta-analysis of Observational Studies in Epidemiology; STROBE: Strengthening the Reporting of Observational Studies in Epidemiology; STARD: Standards for Reporting of Diagnostic Accuracy; SQUIRE: Standards for Quality Improvement Reporting Excellence; QUOROM: Quality of Reporting of Meta-analyses; MIAME: Minimum Information About a Microarray Experiment; MIBBI: Minimum Information for Biological and Biomedical Investigations; ARRIVE: Animal Research: Reporting *In Vivo* Experiments

The availability of reporting guidelines by itself is not sufficient to drive improvements in the completeness of reporting: journal practices clearly have a major role to play [26, 27]. However, the extent to which the journals endorse and adhere to the reporting guidelines seems to vary widely [28]. The most well-known reporting guideline, CONSORT, has been adopted by 585 biomedical journals, and empirical evidence supports the view that the implementation of CONSORT has been associated with improved reporting of randomized trials [1, 29]. This has been achieved over an extensive period of nearly two decades (CONSORT was initially introduced in 1996). Given such timescales in the uptake of reporting guidelines by journals, sufficient time should be allowed prior to assessing the level of awareness of any new guideline such as STRICTA [30]. There might be intrinsic challenges for STRICTA to obtain a degree of support comparable to CONSORT, as it is a ‘specialty-specific’ reporting guideline not applicable for all journals; this notwithstanding, it follows that more strict enforcement of STRICTA within the endorsed journals is more likely to yield the benefits for which it was intended.

### Citation of STRICTA

As fourteen years have elapsed since STRICTA were first published, we think it is timely to examine the utilization and potential impacts of STRICTA.

Although it is possible to measure the impact of STRICTA in numerous ways, perhaps the most logical (if novel) approach is examination of citations for STRICTA. Furthermore a broad search of the literature has shown that previous reviews of STRICTA have been limited to English-language papers (and papers published in English) [1]. We therefore decided to conduct a comprehensive evaluation of the application of STRICTA in relevant articles in three publication languages - English, Chinese, and Japanese. The latter two are considered representative of the main acupuncture research in Asian countries.

In our approach we used Cited Reference Search and/or Citation Tracker (slight differences in different databases) for two English databases (*Web of Science* and *Scopus*), one Chinese database (*China National Knowledge Infrastructure*) and one Japanese database (*Japan Science and Technology Information Aggregator, Electronic*) to explore citations for STRICTA 2002 and STRICTA 2010 from 2002 up to January 2015. For the literature in English, full titles of publications were used as search terms. Due to potential limitations on the dissemination of the English STRICTA in non-English speaking countries, the key word and/or the topic of “STRICTA” were used as additional search terms in both Chinese and Japanese databases. Two standardized spreadsheets were used to record the basic characteristics of articles regarding the publication year, study type, and the journal type (general medical non-CAM journals, specialty medical non-CAM journals, CAM journals with STRICTA endorsement, and CAM journals without STRICTA endorsement) [1]. We calculated the annual STRICTA citation counts, including those relating exclusively to RCTs and related studies (e.g. protocols) for which STRICTA was initially designed. We then set up line plots to investigate the possible changes in citations over time. Linear regression (the Spearman’s rank correlation) was used to identify the potential predictors of citation counts, i.e. the language of publication, and the year of publication.

Our search strategy resulted in the identification of 519 articles that cited STRICTA 2002 and 351 that cited STRICTA 2010, and a further 47 articles in Chinese and 11 in Japanese that mentioned STRICTA as either a key word or topic. After excluding duplicates and those outside of the study limitations, a total of 536 papers were eligible for inclusion. The main characteristics of the articles are displayed in (Additional file 1: Table S1 and Additional file 2: Table S2).

**Fig. 1:**
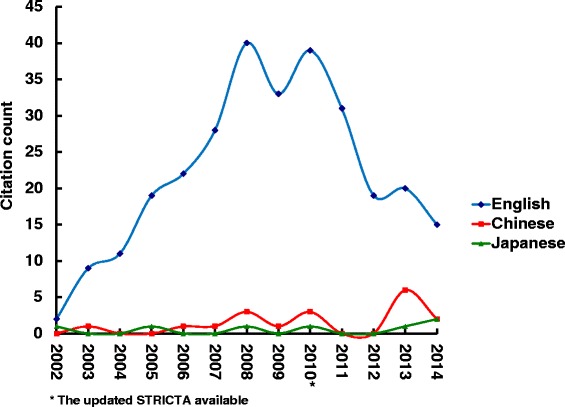
Citation of STRICTA 2002 in all studies

**Fig. 2:**
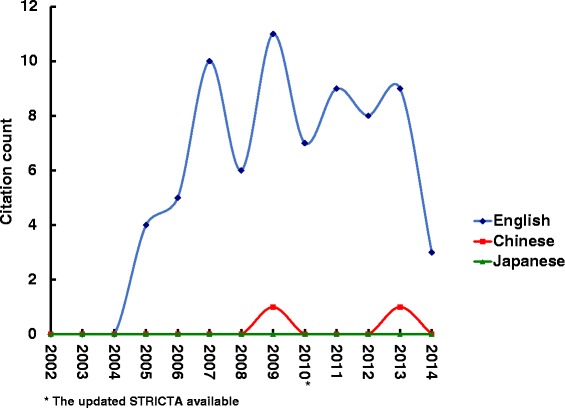
Citation of STRICTA 2002 in RCTs

**Fig. 3:**
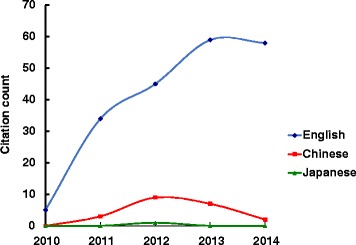
Citation of STRICTA 2010 in all studies

**Fig. 4:**
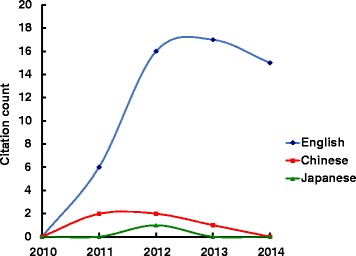
Citation of STRICTA 2010 in RCTs

**Fig. 5:**
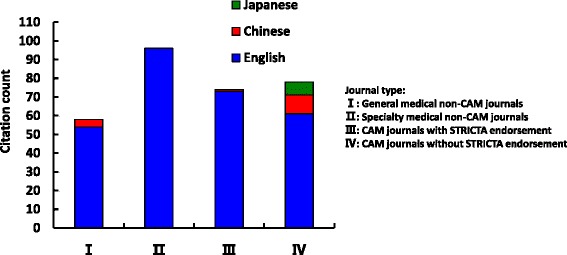
Publication location of articles citing STRICTA 2002

**Fig. 6:**
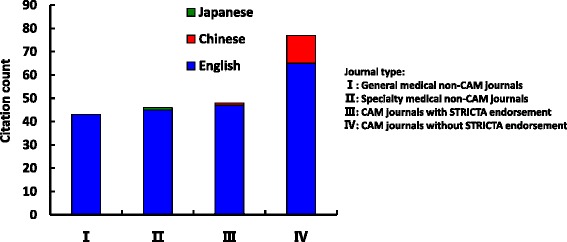
Publication location of articles citing STRICTA 2010

Overall citation counts increased over time. For the STRICTA guidelines 2002, the two English databases demonstrated growing citation numbers until the revised STRICTA were published in 2010: it grew steadily from two citations in 2002 to 40 citations in 2008, but plateaued over the next two years (Fig. 1). In the context of citations in RCTs, this similarly increased with some oscillation from 2005 (three years after the initial introduction); however, the counts were low (maximum of 11 citations in 2009) (Fig. 2). For the STRICTA guidelines 2010, continuing citation was found for both RCTs and papers of other study types published in English (Figs. 3 and 4). By contrast, in the Chinese literature there were fewer citations. Although there were small increases (with some small variation), few authors referred to the STRICTA guidelines in their publications: until the end of 2014, the STRICTA 2002 was cited in total only 18 times and the STRICTA 2010 21 times; only seven such RCTs have cited STRICTA over the past 14 years. For citation in Japanese articles, STRICTA 2002 and STRICTA 2010 were barely cited: seven articles cited the STRICTA guidelines 2002 (published in the years 2002, 2005, 2008, 2010, 2013 and 2014, respectively), and only one RCT article (2012) cited the revised STRICTA 2010. Results of linear regression suggest that the date of publication was a significant predictor of increasing citation counts for STRICTA guidelines 2010 in English literature (r = 0.900). With regard to journal of publication, authors citing STRICTA 2002 preferred publishing the articles in non-CAM specialty medical journals in the English language, whilst those published in Chinese and Japanese languages were in the CAM journals without STRICTA endorsement (Fig. 5). In contrast, for STRICTA 2010, the most frequently cited were those published in the CAM journals without STRICTA endorsement for both English and Chinese languages (Fig. 6).

### Implications for STRICTA

Since the first systematic review which examined the impact of STRICTA on the reporting quality of acupuncture trials [1], subsequent evaluations have drawn similar conclusions: it appears that STRICTA (2002 and 2010) has not improved the quality of reporting to the degree anticipated, thus descriptions of the acupuncture specific aspects of publications remain suboptimal [3–5]. Although a small increase (17 %) in reporting quality was revealed by another review using a combined CONSORT and STRICTA assessment instrument [2], the reporting of practitioner background (e.g. training, experience and expertise) is extremely poor, and there continues to be a need for improvement in reporting on the details of acupuncture trials [1, 2]. Besides potential lack of awareness of the guidelines based on the limited numbers found in the database searches [1], it is possible that dissemination of the STRICTA guidelines was a limiting factor in itself, though we acknowledge the means of dissemination are clearly outlined on the web site.

In regard to potential for improvements with respect to the dissemination and use of STRICTA (2010) the following concepts could be considered. Firstly, it may be beneficial to update the STRICTA checklist. As an official extension of the CONSORT statement 2010 (item 5), the content integrity of the STRICTA guidelines may be improved by embedding all the items suggested by the TIDieR guidelines (which specify a minimum set of items to describe an intervention) in the checklist. For the STRICTA to remain credible it may be helpful to collaborate with the TIDieR group, and to refer to the other relevant reporting guidelines in the EQUATOR network. The revision process might also benefit from solicitation of a wider range of expert opinions. It is argued by Chinese scholars that several Traditional Chinese Medicine characteristics of acupuncture (e.g. syndrome differentiations, treatment opportunities, and disease stages) have not been clearly articulated in STRICTA [31]. Furthermore, it is also suggested that items concerning the standards and precision of acupoint localizations, the angle and direction of needle insertion, and the acupuncture experiences of participants are included in any future revision of STRICTA [31–33]. Furthermore we suggest the updated STRICTA checklist be standardized with the current format and any modifications or additions clearly highlighted.

A second strategy may be to improve the efficiency of reporting by using a flowchart. As print journals have limitations on manuscript length, full reporting of all intervention details within one primary paper is almost impossible, especially when the study protocols or relevant papers have not been previously published. Although hosting the specific components in relevant websites may seem to be a rational solution, there are inherent risks such as resourcing, and responsibility for maintaining the website. Such problems were revealed in a recent review which evaluated the description of non-pharmacological interventions in randomized trials [34]. A more fruitful approach might be to provide a graphical representation instead of text descriptions of the details of the interventions. This might be based on a flowchart with squares and circles which represent the objects and activities of each treatment arms in specific columns; below the flowchart, a legend could provide brief instructions of each components, including forms, contents, functions, and details of the intervention providers [35]. Such an approach may clarify the majority of components (except for the rationale of interventions) required by the STRICTA checklist. In addition, it may have advantages and encourage the use of guidelines more rigorously including: providing a better description of the interventions, decreasing manuscript word count, promoting an easier understanding of the trial structures, and helping with the design and conduct of acupuncture research at the early stage [36].

Thirdly, there is potential for journals that endorse STRICTA to be more consistent with their requirements for authors to adhere to the Guidelines. The benefits of reporting in accordance with the guidelines, and in publications that use the guidelines was reinforced by Hopewell *et al* [37] in their review of the CONSORT statement. Likewise for STRICTA, we advocate for more consistent enforcement of adherence to the guidelines. There continues to be a need for complete descriptions in clinical trials of acupuncture. Thus it may be advantageous for the STRICTA group to develop an official statement with a clear message relating to adherence requirements. One possible way forward would be to request authors to comply with reporting on specific details and submit a completed STRICTA checklist as a requirement for consideration for publication [37, 38]. This may be assisted in the future by using web-based technologies that allow automated text identification for essential elements [39]. We suggest the statement be included in the published “*Instructions to Authors*” for the relevant journal. In addition, an appropriate reference to the STRICTA guidelines and an accessible URL of the STRICTA web site, as author support resources would also be expected to be provided by the journal. As it is argued by some that reviewers and editors unfamiliar with STRICTA may be responsible for the missing details relating to acupuncture in the final publications [12], health related journals would need to ensure their editorial staff enforce adherence to STRICTA, and incorporate the checklist into the peer-review and editorial processes. In addition, journals may consider providing editorials to encourage use, the positive impacts of which have been demonstrated by the greater degree of CONSORT implementation [38, 40].

Although STRICTA is designed mainly for clinical trials, its application may also be usefully applied to systematic reviews. The reporting quality of RCTs has been shown to be better than that of systematic reviews [41], and for Cochrane reviews of acupuncture, the reporting of treatment details has been noted to be inadequate [30]. As few reviews recommend appropriate treatment regimen(s) to be used in routine practice, practitioners in such cases are unable to use the related evidence to inform their treatments [41]. Thus, it may be beneficial for journals to widen the use of STRICTA to acupuncture reviews, which would improve the applicability and utilization of review results in future research and clinical practice.

Fourthly, as there is evidence of a lack of consistency among journals not published in the English language, it may be beneficial to implement a strategy where the Guidelines are more strictly endorsed in non-English speaking countries. Our findings showed growing levels of uptakes of STRICTA in the English literature, but the citations of STRICTA in Asian articles, such as Chinese and Japanese, appeared inconsistent and remain low. It seems that other strategies besides the STRICTA translations are worthy of consideration. As there is a large amount of acupuncture research undertaken in non-English-speaking countries, for example in China, Japan, Korea, and Germany, it may be feasible to develop a global network of STRICTA research centers. Possible missions of these centers would be to organize regular education and training workshops or seminars (including webinars) which concentrate on the efficient use of the STRICTA guidelines and good research reporting practices, seek users’ feedback, handle criticism from all stakeholders, and maximize endorsements and adherence to STRICTA in local journals.

Fifthly, consideration could be given to updating and expanding the STRICTA website. Our findings show that details on key elements of the development procedure of the STRICTA guidelines, such as the pre-meeting activities and the face-to-face consensus meeting, is not publicly available (or is hard to locate). This finding concurred with the previous systematic review by Moher * et al* in which the authors noted the problems of non-transparent processes for the development of, and reporting on, guidelines [42]. It may be possible to provide the update data of STRICTA on the web site, and append this address in articles on the Guidelines. Beyond this, related papers that have evaluated the reporting quality of the acupuncture studies could be included on the web site, to inform the impact of STRICTA, and to help maintain it. It is expected that such evaluations could be undertaken collaboratively by different research parties including authors, editors, publishers and funders. This may reduce the risk of bias in self-evaluations, and also provide a better opportunity for broader support [43]. It may also be useful to include a “News” section concerning any new issues related to the guidelines such as plans for updates, and perhaps the STRICTA centers, if possible.

Finally, we recommend providing the linkage to the EQUATOR network (http://www.equator-network.org) [22], a repository for good reporting guidelines for health research, on the STRICTA web site, to improve the general standards of reporting of research within the field of acupuncture.

## Summary

A complete description of complex interventions is challenging [23]; the process of treatment such as specific regimens and description of the necessary materials, are often missing from such descriptions [41]. Problems with this inadequate reporting have led to the creation of several reporting guidelines, including STRICTA and those in other areas such as homeopathy, yoga, and moxibustion [44–46]. Although it is encouraging to note that the development of these reporting guidelines may be one valuable step towards the enhancement of reporting quality and research reliability, it is only a first step. Given the low utilization, and consequently the limited impact, of these guidelines, specific implementation strategies are necessary to remedy this situation.

We believe that the quality of reporting of the interventions in clinical trials of acupuncture will only be improved by wide dissemination and strict adherence to the STRICTA guidelines. Beyond the current implementation measures such as co-publications and translations into other languages, more effort is needed to disseminate and promote the more widespread use of STRICTA. All stakeholders (i.e. researchers, journal publishers, funding organizations, policy regulators) should take a shared responsibility to collaborate to implement STRICTA to a greater extent. This will, in turn, ensure the quality of reports of acupuncture studies is improved, thus assisting the potential for research results to be efficiently translated into clinical practice. As there is a lack of sufficient funding for developing reporting guidelines [43], we particularly appeal to the health research funding organizations to emphasize the importance of STRICTA, and ensure dedicated funding to support its revision and maintenance on a routine basis in future. Such support is believed to influence reporting quality and, therefore, the subsequent impact, of future acupuncture research.

It may be argued by some that if the reporting guidelines themselves are deficient, this would undermine their impact on improvement in scientific quality and thus the benefits to clinical practice. However, it may also be argued that improvements in the reporting guidelines are based on their wide utilization and exposure to critical appraisal. Continual improvement in the quality of reporting related to particular treatment interventions will also benefit clinical practice and drive research further forward.

As yet there are no formal tools to assess the validity of the reporting guidelines; the *Guidance for Developers of Health Research Reporting Guidelines* [21] and the *TIDieR* [23] are designed to inform the development of reporting guidelines. We used the original format of the *TIDieR*, and kept the 18 primary items of the *Guidance for Developers of Health Research Reporting Guidelines*. Our two validity checklists are helpful but do have limitations, as some items are optional and there is no weighting criteria of individual items. Hence, in order to reduce bias, we call for more researchers to participate in the evaluation, provide critical appraisements, and help with the optimization of the STRICTA guidelines.

## Additional files

Additional file 1: Table S1. Characteristics of articles cited STRICTA 2002. The table presents the characteristics of the articles that cited the STRICTA guidelines 2002, including the title, author(s), publication year and journal, study type, and journal type, of the articles. Additional file 2: Table S2. Characteristics of articles cited STRICTA 2010. The table presents the characteristics of the articles that cited the STRICTA guidelines 2010, including the title, author(s), publication year and journal, study type, and journal type, of the articles.

## Abbreviations

STRICTA: Standards for Reporting Interventions in Controlled Trials of Acupuncture
RCTs: Randomized controlled trials
CAM: Complementary and Alternative Medicine
CONSORT: Consolidated Standards of Reporting Trials
EQUATOR: Enhancing the Quality and Transparency of Health Research
TIDieR: Template for Intervention Description and Replication
