# Supplementary material for: STRICTA: is it time to do more?
Source: BMC Complement Altern Med. 2015 Jun 20;15:190. doi: 10.1186/s12906-015-0714-4 (PMC4474462; doi:10.1186/s12906-015-0714-4)
Supplement: Additional file 1: Table S1. — Characteristics of articles cited STRICTA 2002. The table presents the characteristics of the articles that cited the STRICTA guidelines 2002, including the title, author(s), publication year and journal, study type, and journal type, of the articles. [file 12906_2015_714_MOESM1_ESM.pdf]

Table S1. Characteristics of articles cited STRICTA 2002

Publication year	Author(s)	Article title	Study type	Journal name	Journal type
From English databases (Web of Science and Scopus)					
2002	Niemtzow	Acupuncture in the twenty-first century	Editorial	Journal of Alternative and Complementary Medicine	3
2002	Webster-Harrison et al	Acupuncture for tennis elbow: An e-mail consensus study to define a standardised treatment in a GPs' surgery	Delphi study	Acupuncture in Medicine	3
2003	Anastasi and McMahon	Testing strategies to reduce diarrhea in persons with HIV using traditional Chinese medicine: Acupuncture and moxibustion	Observational study	Journal of the Association of Nurses in AIDS Care	2
2003	Birch	Overview of models used in controlled acupuncture studies and thoughts about questions answerable by each	Review: research methodology discussions	Clinical Acupuncture and Oriental Medicine	4
2003	Dusek et al	Healing prayer outcomes studies: Consensus recommendations	Recommendations	Alternative Therapies in Health and Medicine	4
2003	Hammerschlag	Acupuncture: On what should its evidence base be based?	Commentary	Alternative Therapies in Health and Medicine	4
2003	Streitberger et al	Quality standards for randomised controlled acupuncture trials (Article in German)	Review: research methodology discussions	Deutsche Zeitschrift für Akupunktur	3
2003	Usichenko and Pavlovic	Suggesting the optimal control procedure for acupressure studies	Letter	Anesthesia and Analgesia	2
2003	White and Ernst	Pitfalls in conducting systematic reviews of acupuncture	Letter	Rheumatology	2
2003	Wiebrecht	Recommendations of the standards for reporting interventions in controlled trials of acupuncture (STRICTA) (Article in German)	Recommendations	Deutsche Zeitschrift für Akupunktur	3
2003	Zijlstra et al	Anti-inflammatory actions of acupuncture	Review	Mediators of Inflammation	2

2004	Audette and Ryan	The role of acupuncture in pain management	Review	Physical Medicine and Rehabilitation Clinics of North America	2
2004	Birch	Clinical research on acupuncture: Part 2. Controlled clinical trials, an overview of their methods	Review: research methodology discussions	Journal of Alternative and Complementary Medicine	3
2004	Cardini	Acupuncture: Scientific evidence	Review	Evidenze scientifiche in agopuntura	4
2004	Long et al	Original research published in the chiropractic literature: Evaluation of the research report	Review: research methodology discussions	Journal of Manipulative and Physiological Therapeutics	2
2004	MacPherson	Pragmatic clinical trials	General article: expert opinion	Complementary Therapies in Medicine	3 (2002)
2004	McCarney et al	An overview of two Cochrane systematic reviews of complementary treatments for chronic asthma: Acupuncture and homeopathy	Review	Respiratory Medicine	2
2004	Peuker and Filler	Guidelines for case reports of adverse events related to acupuncture	Guidelines	Acupuncture in Medicine	3
2004	Rogenhofer et al	Acupuncture for pain in extracorporeal shockwave lithotripsy	Observational study	Journal of Endourology	2
2004	Veal	Countering misleading information	Review: research methodology discussions	Complementary Therapies in Nursing and Midwifery	4
2004	White	Writing case reports - Author guidelines for Acupuncture in Medicine	Guidelines	Acupuncture in Medicine	3
2004	White	Methodological concerns when designing trials for the efficacy of acupuncture for the treatment of pain	Review: research methodology discussions	Complementary and Alternative Approaches to Biomedicine	4
2005	Altman	The development of the CONSORT explanatory document	Guidelines related	Chinese Journal of Evidence-Based Medicine	1
2005	Altman and Moher	Developing guidelines for reporting healthcare research: Scientific rationale and procedures	Guidelines related	Medicina Clinica	1

2005	Baldry and Thompson	Acupuncture, Trigger Points and Musculoskeletal Pain	Book	Publisher: Elsevier Ltd	NA
2005	Castro	Integrating acupuncture in the physical medicine and rehabilitation setting	Review	Critical Reviews in Physical and Rehabilitation Medicine	2
2005	Furlan et al	Acupuncture and dry-needling for low back pain: An updated systematic review within the framework of the Cochrane Collaboration	Review	Spine	2
2005	Furlan et al	Acupuncture and dry-needling for low back pain	Review	Cochrane Database of Systematic Reviews	1
2005	Herman et al	Is complementary and alternative medicine (CAM) cost-effective? A systematic review	Review	BMC Complementary and Alternative Medicine	4
2005	Jedel	Acupuncture in xerostomia - A systematic review	Review	Journal of Oral Rehabilitation	2
2005	Johnston et al	Incorporating acupuncture into oncologic practice: How clinical need shapes physician acceptance	Commentary	Journal of Cancer Integrative Medicine	2
2005	Lechleitner et al	Feasibility and acceptance of acupuncture treatment in a Department of Internal Medicine (Article in German)	Observational study	Deutsche Zeitschrift für Akupunktur	3
2005	Melchart et al	The acupuncture randomised trial (ART) for tension-type headache - Details of the treatment	RCT	Acupuncture in Medicine	3
2005	Mills et al	An analysis of general medical and specialist journals that endorse CONSORT found that reporting was not enforced consistently	Review: research methodology discussions	Journal of Clinical Epidemiology	1
2005	Mills et al	The quality of randomized trial reporting in leading medical journals since the revised CONSORT statement	Review: research methodology discussions	Contemporary Clinical Trials	1
2005	Rathbone and Xia	Acupuncture for schizophrenia	Review	Cochrane Database of Systematic Reviews	1
2005	Smith and Hay	Acupuncture for depression	Review	Cochrane Database of Systematic Reviews	1
2005	Thomas et al	Longer term clinical and economic benefits of offering acupuncture care to patients with chronic low back pain	RCT	Health Technology Assessment	1

2005	Usichenko et al	Auricular acupuncture for pain relief after total hip arthroplasty - A randomized controlled study	RCT	Pain	2
2005	Usichenko et al	Auricular acupuncture for pain relief after ambulatory knee arthroscopy - A pilot study	RCT	Evidence-based Complementary and Alternative Medicine	4
2005	White	Conducting and reporting case series and audits - Author guidelines for Acupuncture in Medicine	Guidelines	Acupuncture in Medicine	3
2006	Allen et al	Acupuncture for depression: A randomized controlled trial	RCT	Journal of Clinical Psychiatry	2
2006	Brinkhaus et al	Interventions and physician characteristics in a randomized multicenter trial of acupuncture in patients with low-back pain	RCT	Journal of Alternative and Complementary Medicine	3
2006	Dean et al	Reporting Data on Homeopathic Treatments (RedHot): A supplement to CONSORT	Guideline	Forschende Komplementarmedizin	4
2006	Gagnier et al	Recommendations for reporting randomized controlled trials of herbal interventions: explanation and elaboration	Recommendations	Journal of Clinical Epidemiology	2
2006	Griggs and Jensen	Effectiveness of acupuncture for migraine: Critical literature review	Review	Journal of Advanced Nursing	2
2006	Highfield et al	Adolescent endometriosis-related pelvic pain treated with acupuncture: Two case reports	Case report	Journal of Alternative and Complementary Medicine	3
2006	Johnson	The clinical effectiveness of acupuncture for pain relief - You can be certain of uncertainty	Review	Acupuncture in Medicine	3
2006	Kessler et al	A study to compare the effects of massage and static touch on experimentally induced pain in healthy volunteers	Observational study	Physiotherapy	2
2006	Lee and Chan	Acupuncture and anaesthesia	Review	Best Practice and Research: Clinical Anaesthesiology	2
2006	Lee et al	Acupuncture for smoking cessation?: Commentary	Commentary	Yonsei Medical Journal	1
2006	Lewis and Johnson	The clinical effectiveness of therapeutic massage for musculoskeletal pain: a systematic review	Review	Physiotherapy	2
2006	Lewith et al	Developing a research strategy for acupuncture	Review: research methodology discussions	Clinical Journal of Pain	2

2006	Lim et al	Acupuncture for treatment of irritable bowel syndrome	Review	Cochrane Database of Systematic Reviews	1
2006	Linde et al	Treatment in a randomized multicenter trial of acupuncture for migraine (ART migraine)	RCT	Forschende Komplementarmedizin und Klassische Naturheilkunde	4
2006	Peters	Letter to the editor	Letter	Journal of Bodywork and Movement Therapies	2
2006	Price et al	Acupuncture care for breast cancer patients during chemotherapy: A feasibility study	Protocol	Integrative Cancer Therapies	2
2006	Shea	Applying evidence-based medicine to traditional Chinese medicine: Debate and strategy	Review: research methodology discussions	Journal of Alternative and Complementary Medicine	3
2006	Smith et al	Complementary and alternative therapies for pain management in labour	Review	Cochrane Database of Systematic Reviews	1
2006	Usichenko et al	Auricular acupuncture reduces intraoperative fentanyl requirement during hip arthroplasty - A randomized double-blinded study	RCT	Acupuncture and Electro-Therapeutics Research	4
2006	Walji and Boon	Redefining the randomized controlled trial in the context of acupuncture research	Review: research methodology discussions	Complementary Therapies in Clinical Practice	4
2006	Wu et al	Acupuncture for stroke rehabilitation	Review	Cochrane Database of Systematic Reviews	1
2006	Zhang et al	Assessing the reporting quality of randomized controlled trials on acupuncture for acute ischemic stroke using the CONSORT statement and STRICTA	Review: research methodology discussions	Chinese Journal of Evidence-Based Medicine	1
2007	Anderson et al	In vitro fertilization and acupuncture: Clinical efficacy and mechanistic basis	Review	Alternative Therapies in Health and Medicine	4
2007	Banzer et al	Acute effects of needle acupuncture on power performance during strech-shortening cycle (Article in German)	RCT	Forschende Komplementarmedizin	4
2007	Barlas	Is all temporomandibular joint pain trigger point related and can acupuncture really help? Commentary	Commentary	Focus on Alternative and Complementary Therapies	4

2007	Brinkhaus	The effect of acupuncture and probiotics in children with asthma: Commentary	Commentary	Focus on Alternative and Complementary Therapies	4
2007	Brinkhaus and Cabrini	Does acupuncture reduce anxiety in patients undergoing cataract surgery? Commentary	Commentary	Focus on Alternative and Complementary Therapies	4
2007	Brinkhaus et al	Physician and treatment characteristics in a randomised multicentre trial of acupuncture in patients with osteoarthritis of the knee	RCT	Complementary Therapies in Medicine	3 (2002)
2007	Crew et al	Pilot study of acupuncture for the treatment of joint symptoms related to adjuvant aromatase inhibitor therapy in postmenopausal breast cancer patients	RCT	Journal of Cancer Survivorship	2
2007	Dean et al	Reporting data on homeopathic treatments (RedHot): a supplement to CONSORT	Guideline	Homeopathy	4
2007	Dean et al	Reporting data on homeopathic treatments (RedHot): a supplement to CONSORT	Guideline	Journal of Alternative and Complementary Medicine	3
2007	Dhond et al	Neuroimaging acupuncture effects in the human brain	Review	Journal of Alternative and Complementary Medicine	3
2007	Ezzo et al	Massage for mechanical neck disorders: A systematic review	Review	Spine	2
2007	Fassoulaki et al	Acupressure on the extra 1 acupoint: The effect on bispectral index, serum melatonin, plasma β-endorphin, and stress	RCT	Anesthesia and Analgesia	2
2007	Hansson et al	Intramuscular and periosteal acupuncture for anxiety and sleep quality in patients with chronic musculoskeletal pain - An evaluator blind, controlled study	RCT	Acupuncture in Medicine	3
2007	Harbach et al	Minimal immunoreactive plasma β-endorphin and decrease of cortisol at standard analgesia or different acupuncture techniques	RCT	European Journal of Anaesthesiology	2
2007	Julliard et al	Towards a model for planning clinical research in oriental medicine	Review: research methodology discussions	Explore: The Journal of Science and Healing	1

2007	Kalyon	Acupuncture therapy (Article in Turkish)	Review	Turkiye Fiziksel Tip ve Rehabilitasyon Dergisi	2
2007	Kvist et al	A randomised-controlled trial in Sweden of acupuncture and care interventions for the relief of inflammatory symptoms of the breast during lactation	RCT	Midwifery	2
2007	Leo and Ligot	A systematic review of randomized controlled trials of acupuncture in the treatment of depression	Review	Journal of Affective Disorders	2
2007	Liou et al	Broken needle in the cervical spine: A previously unreported complication of Xiaozendao acupuncture therapy	Case report	Journal of Alternative and Complementary Medicine	3
2007	Lu et al	Acupuncture for chemotherapy-induced leukopenia: Exploratory meta-analysis of randomized controlled trials	Review	Journal of the Society for Integrative Oncology	2
2007	MacPherson and Schroer	Acupuncture as a complex intervention for depression: A consensus method to develop a standardised treatment protocol for a randomised controlled trial	Delphi study	Complementary Therapies in Medicine	3 (2002)
2007	Prady and MacPherson	Assessing the utility of the Standards for Reporting Trials of Acupuncture (STRICTA): A survey of authors	Survey	Journal of Alternative and Complementary Medicine	3
2007	Rosenthal and Anderson	Acupuncture and in vitro fertilisation: Recent research and clinical guidelines	Review	Journal of Chinese Medicine	4
2007	Selmer-Olsen et al	Does acupuncture used in nulliparous women reduce time from prelabour rupture of membranes at term to active phase of labour? A randomised controlled trial	RCT	Acta Obstetricia Et Gynecologica Scandinavica	2
2007	Usichenko et al	Auricular acupuncture for pain relief after ambulatory knee surgery: A randomized trial	RCT	Canadian Medical Association Journal	1
2007	Walach	RedHot	Letter	Contemporary Clinical Trials	1

2007	Wang et al	Effect of acupuncture-like electrical stimulation on chronic tension-type headache: A randomized, double-blinded, placebo-controlled trial	RCT	Clinical Journal of Pain	2
2007	White et al	Standardization of nomenclature in acupuncture research (SoNAR)	Recommendations	Evidence-based Complementary and Alternative Medicine	4
2008	Balk	Why we should change the course of acupuncture research	Review: research methodology discussions	Journal of Chinese Medicine	4
2008	Baxter et al	Clinical effectiveness of laser acupuncture: A systematic review	Review	Journal of Acupuncture and Meridian Studies	4
2008	Colbert et al	Magnets applied to acupuncture points as therapy - A literature review	Review	Acupuncture in Medicine	3
2008	Conn et al	Searching for the intervention in intervention research reports	Review: research methodology discussions	Journal of Nursing Scholarship	2
2008	Cui et al	Acupuncture for restless legs syndrome	Review	Cochrane Database of Systematic Reviews	1
2008	Donnellan and Sharley	Comparison of the effect of two types of acupuncture on quality of life in secondary progressive multiple sclerosis: a preliminary single-blind randomized controlled trial	RCT	Clinical Rehabilitation	2
2008	Ee et al	Acupuncture for pelvic and back pain in pregnancy: a systematic review	Review	American Journal of Obstetrics and Gynecology	2
2008	Enblom et al	Can individuals identify if needling was performed with an acupuncture needle or a non-penetrating sham needle?	RCT	Complementary Therapies in Medicine	3 (2002)
2008	Fleckenstein	A systematic review of the clinical effectiveness of acupuncture for allergic rhinitis (Article in German)	Note	Deutsche Zeitschrift für Akupunktur	3
2008	Hammerschlag et al	Acupuncture Research	Book Chapter	Publisher: Elsevier Ltd	NA
2008	Hopwood et al	Evaluating the efficacy of acupuncture in defined aspects of stroke recovery: A randomised, placebo controlled single blind study	RCT	Journal of Neurology	2

2008	Jeon et al	Proteomic analysis of the neuroprotective mechanisms of acupuncture treatment in a Parkinson's disease mouse model	Animal studies	Proteomics	2
2008	Lam et al	Efficacy and safety of acupuncture for idiopathic Parkinson's disease: A systematic review	Review	Journal of Alternative and Complementary Medicine	3
2008	Lau and Jones	A single session of Acu-TENS increases FEV1 and reduces dyspnoea in patients with chronic obstructive pulmonary disease: A randomised, placebo-controlled trial	RCT	Australian Journal of Physiotherapy	2
2008	Lee	Acupuncture and acupressure for post-chemotherapy fatigue - We have only just begun: Commentary	Commentary	Focus on Alternative and Complementary Therapies	4
2008	Lee et al	Auricular acupuncture for insomnia: A systematic review	Review	International Journal of Clinical Practice	1
2008	Lee et al	Acupuncture versus sham acupuncture for chronic prostatitis/chronic pelvic pain	RCT	American Journal of Medicine	1
2008	Lu et al	The value of acupuncture in cancer care	Review	Hematology/Oncology Clinics of North America	2
2008	MacPherson et al	Developments in acupuncture research: Big-picture perspectives from the leading edge	Conference paper	Journal of Alternative and Complementary Medicine	3
2008	McDonough and McNeill	Acupuncture	Book Chapter	Book: Complementary Therapies for Physical Therapy	NA
2008	Molsberger et al	Chinese acupuncture for chronic low back pain: An international expert survey	Survey	Journal of Alternative and Complementary Medicine	3
2008	Myers et al	Massage modalities and symptoms reported by cancer patients: Narrative review	Review	Journal of the Society for Integrative Oncology	2
2008	Prady et al	A systematic evaluation of the impact of STRICTA and CONSORT recommendations on quality of reporting for acupuncture trials	Review: research methodology discussions	PLoS ONE	1
2008	Reider	Acronyms and anachronisms	Editorial	American Journal of Sports Medicine	2

2008	Rooney	Acupuncture in the treatment of non-specific low back pain in an adult population: A review of the evidence	Review	Internet Journal of Advanced Nursing Practice	2
2008	Sagar	Acupuncture as an evidence-based option for symptom control in cancer patients	Review	Current Treatment Options in Oncology	2
2008	Sagar and Wong	Research and regulatory issues for integrative oncology	Review: research methodology discussions	Current Oncology	2
2008	Simera et al	Guidelines for reporting health research: The EQUATOR network's survey of guideline authors	Survey	PLoS Medicine	3
2008	Stener-Victorin et al	Acupuncture in polycystic ovary syndrome: Current experimental and clinical evidence	Review	Journal of Neuroendocrinology	2
2008	Stener-Victorin and Von Hagens	Acupuncture in assisted reproductive technology and PCOS	Review	Gynakologische Endokrinologie	2
2008	Usichenko	Pain treatment with methods of complementary medicine	Conference paper	Journal fur Anasthesie und Intensivbehandlung	2
2008	Wang et al	Acupuncture for pain relief in patients with rheumatoid arthritis: A systematic review	Review	Arthritis & Rheumatism- Arthritis Care & Research	2
2008	Wayne et al	Increasing research capacity at the New England School of Acupuncture through faculty and student research training initiatives	General article: education columns	Alternative Therapies in Health and Medicine	4
2008	Wayne and Kaptchuk	Challenges inherent to t'ai chi research: Part II - Defining the intervention and optimal study design	Review: research methodology discussions	Journal of Alternative and Complementary Medicine	3
2008	White et al	Defining an adequate dose of acupuncture using a neurophysiological approach - A narrative review of the literature	Review	Acupuncture in Medicine	3
2008	Xie et al	Acupuncture for dysphagia in acute stroke	Review	Cochrane Database of Systematic Reviews	1
2008	Yeh	Commentary on the Cochrane review of Tai Chi for rheumatoid arthritis	Commentary	Explore: The Journal of Science and Healing	1

2008	Yuan et al	Treatment regimens of acupuncture for low back pain-A systematic review	Review	Complementary Therapies in Medicine	3 (2002)
2008	Yuan et al	Effectiveness of acupuncture for low back pain: A systematic review	Review	Spine	2
2008	Zheng et al	The effect of electroacupuncture on opioid-like medication consumption by chronic pain patients: A pilot randomized controlled clinical trial	RCT	European Journal of Pain	2
2009	Anastasi et al	Symptom management for irritable bowel syndrome: A pilot randomized controlled trial of acupuncture/moxibustion	RCT	Gastroenterology Nursing	2
2009	Andrade and Radhakrishnan	Prayer and healing: A medical and scientific perspective on randomized controlled trials	Review	Indian Journal of Psychiatry	2
2009	Bai et al	Spatiotemporal modulation of central neural pathway underlying acupuncture action: A systematic review	Review	Current Medical Imaging Reviews	1
2009	Benham and Johnson	Could acupuncture needle sensation be a predictor of analgesic response?	Commentary	Acupuncture in Medicine	3
2009	Borud et al	The Acupuncture on Hot Flushes Among Menopausal Women (ACUFLASH) study, a randomized controlled trial	RCT	Menopause-the Journal of the North American Menopause Society	2
2009	Brinkhaus	Effectiveness of diet and exercise vs. acupuncture for weight loss in obese women: Results of a pilot study - Commentary	Commentary	Focus on Alternative and Complementary Therapies	4
2009	Cho et al	Acupuncture for obesity: A systematic review and meta-analysis	Review	International Journal of Obesity	2
2009	Cho and Whang	Acupuncture for alcohol dependence: A systematic review	Review	Alcoholism: Clinical and Experimental Research	2
2009	Collazo Chao	Effectiveness of acupuncture in relieving pain refractory to conventional pharmacological therapy (Article in Spanish)	Observational study	Revista de la Sociedad Espanola del Dolor	2
2009	Dieterle et al	A prospective randomized placebo-controlled study of the effect of acupuncture in infertile patients with severe oligoasthenozoospermia	RCT	Fertility and Sterility	2

2009	Dijkers	Ensuring Inclusion of Research Reports in Systematic Reviews	Review: research methodology discussions	Archives of Physical Medicine and Rehabilitation	2
2009	Ferreira	Diagnostic accuracy of pattern differentiation algorithm based on Chinese medicine theory: A stochastic simulation study	Observational study	Chinese Medicine	4
2009	Hielm-Bjorkman et al	Psychometric testing of the Helsinki chronic pain index by completion of a questionnaire in Finnish by owners of dogs with chronic signs of pain caused by osteoarthritis	Qualitative study	American Journal of Veterinary Research	2
2009	Hughes	"When I first started going I was going in on my knees, but I came out and I was skipping": Exploring rheumatoid arthritis patients' perceptions of receiving treatment with acupuncture	Qualitative study	Complementary Therapies in Medicine	3 (2002)
2009	Jordan and Lewis	Improving the quality of reporting of research studies	Editorial	Musculoskeletal Care	2
2009	Kottow	Could bioethics recommend acupuncture for public health programmes?	Commentary	Acupuncture in Medicine	3
2009	Lee et al	Acupuncture for treating hot flushes in men with prostate cancer: A systematic review	Review	Supportive Care in Cancer	2
2009	Liu et al	An acupuncture meta-analysis for optic atrophy: Seven randomized, controlled trials	Review	Neural Regeneration Research	2
2009	Lu et al	Acupuncture for chemotherapy-induced neutropenia in patients with gynecologic malignancies: A pilot randomized, sham-controlled clinical trial	RCT	Journal of Alternative and Complementary Medicine	3
2009	MacPherson and Altman	Improving the quality of reporting acupuncture interventions: describing the collaboration between STRICTA, CONSORT and the Chinese Cochrane Centre	Guidelines related	Journal of Evidence-Based Medicine	3

2009	Ospina-Díaz	Introduction to acupuncture. Basics and importance for the primary care physician (Article in Spanish)	Review	Semergen	1
2009	Park et al	Evaluation of manual acupuncture at classical and nondefined points for treatment of functional dyspepsia: A randomized-controlled trial	RCT	Journal of Alternative and Complementary Medicine	3
2009	Roberts et al	A systematic review of the clinical effectiveness of acupuncture for allergic rhinitis (Article in Spanish)	Note	Revista Internacional de Acupuntura	4
2009	Scardina et al	The effect of acupuncture on oral microcirculation in healthy volunteers: An exploratory study	RCT	Acupuncture in Medicine	3
2009	Schroer and MacPherson	Acupuncture, or non-directive counselling versus usual care for the treatment of depression: A pilot study	Protocol	Trials	1
2009	Scott et al	Trigger point injections for chronic non-malignant musculoskeletal pain: A systematic review	Review	Pain Medicine	2
2009	Stock-Schroer et al	Reporting experiments in homeopathic basic research (REHBaR) - A detailed guideline for authors	Guidelines	Homeopathy	4
2009	Wesolowski et al	Acupuncture reveals no specific effect on primary auditory cortex: A functional magnetic resonance imaging study	Observational study	NeuroReport	2
2009	Yang et al	Acupuncture in patients with carpal tunnel syndrome a randomized controlled trial	RCT	Clinical Journal of Pain	2
2009	Yeung et al	Traditional needle acupuncture treatment for insomnia: A systematic review of randomized controlled trials	Review	Sleep Medicine	1
2009	Yeung et al	Electroacupuncture for primary insomnia: A randomized controlled trial	RCT	Sleep	2
2009	Zhang et al	Combination of acupuncture and fluoxetine for depression: A randomized, double-blind, sham-controlled trial	RCT	Journal of Alternative and Complementary Medicine	3

2009	Zheng et al	Acupuncture as a treatment for functional dyspepsia: Design and methods of a randomized controlled trial	RCT	Trials	1
2010	Anastasi et al	Acupuncture/moxibustion RCT for distal sensory peripheral neuropathy in HIV/AIDS: Rationale, design, methods, procedure and logistics	Review: research methodology discussions	European Journal of Oriental Medicine	4
2010	Asher et al	Auriculotherapy for pain management: A systematic review and meta-analysis of randomized controlled trials	Review	Journal of Alternative and Complementary Medicine	3
2010	Capili et al	Adverse event reporting in acupuncture clinical trials focusing on pain	Review: research methodology discussions	Clinical Journal of Pain	2
2010	Cho and Hwang	Acupuncture for primary dysmenorrhoea: A systematic review	Review	BJOG: An International Journal of Obstetrics and Gynaecology	2
2010	Cho and Kim	Efficacy of acupuncture in management of premenstrual syndrome: A systematic review	Review	Complementary Therapies in Medicine	3 (2002)
2010	Collazo Chao	Effectiveness of acupuncture therapy for pain relief in patients with fibromyalgia (Article in Spanish)	Observational study	Revista Internacional de Acupuntura	4
2010	Collazo	Acupuncture treatment in headache refractory to conventional therapies (Article in Spanish)	Observational study	Revista de la Sociedad Espanola del Dolor	2
2010	Cotchett et al	Effectiveness of dry needling and injections of myofascial trigger points associated with plantar heel pain: A systematic review	Review	Journal of Foot and Ankle Research	2
2010	Crew et al	Randomized, blinded, sham-controlled trial of acupuncture for the management of aromatase inhibitor-associated joint symptoms in women with early-stage breast cancer	RCT	Journal of Clinical Oncology	2
2010	Gibson et al	Acupuncture for respiratory disorder: what's the point?	Review	Expert Review of Respiratory Medicine	1
2010	Gross et al	Manipulation or mobilisation for neck pain	Review	Cochrane Database of Systematic Reviews	1

2010	Kim et al	Effects of acupuncture on hot flashes in perimenopausal and postmenopausal women-a multicenter randomized clinical trial	RCT	Menopause	2
2010	Kim and Zhu	Acupuncture for essential hypertension	Review	Alternative Therapies in Health and Medicine	4
2010	Kuo et al	Blood flow effect of acupuncture on the human meridian	Observational study	Medical Acupuncture	4
2010	Langhorst et al	Efficacy of acupuncture in fibromyalgia syndrome-a systematic review with a meta-analysis of controlled clinical trials	Review	Rheumatology	2
2010	Lewith	Acupuncture placebos	Review	European Journal of Oriental Medicine	4
2010	List and Axelsson	Management of TMD: Evidence from systematic reviews and meta-analyses	Review	Journal of Oral Rehabilitation	2
2010	MacPherson	Towards better reporting of interventions in clinical trials of acupuncture	Editorial	Journal of Chinese Integrative Medicine	3
2010	MacPherson et al	Revised standards for reporting interventions in clinical trials of acupuncture (STRICTA): Extending the consort statement (Chinese version)	Guidelines	Journal of Chinese Integrative Medicine	3
2010	MacPherson et al	Revised standards for reporting interventions in clinical trials of acupuncture (STRICTA): Extending the consort statement	Guidelines	Acupuncture in Medicine	3
2010	MacPherson et al	Revised standards for reporting interventions in clinical trials of acupuncture (STRICTA): Extending the consort statement	Guidelines	Journal of Alternative and Complementary Medicine	3
2010	MacPherson et al	Revised standards for reporting interventions in clinical trials of acupuncture (STRICTA): Extending the consort statement	Guidelines	PLoS Medicine	3
2010	MacPherson et al	Revised standards for reporting interventions in clinical trials of acupuncture (STRICTA): Extending the consort statement (Chinese version)	Guidelines	Chinese Journal of Evidence-Based Medicine	1

2010	MacPherson et al	Revised standards for reporting interventions in clinical trials of acupuncture (STRICTA): Extending the consort statement	Guidelines	Medical Acupuncture	3
2010	MacPherson et al	Revised standards for reporting interventions in clinical trials of acupuncture (STRICTA): Extending the consort statement	Guidelines	Journal of Evidence-Based Medicine	3
2010	MacPherson and Jobst	Improving the reporting of interventions in clinical trials of acupuncture: The updated and revised STRICTA	Editorial	Journal of Alternative and Complementary Medicine	3
2010	Miao	Clinical critical qualitative evaluation of the selected randomized controlled trials in current acupuncture researches for low back pain	Review: research methodology discussions	Journal of Chinese Integrative Medicine	3
2010	Miao and Miao	Effect of electroacupuncture on the third lumbar transverse process syndrome: A randomized controlled trial	RCT	Medical Acupuncture	3
2010	Miller et al	Manual therapy and exercise for neck pain: A systematic review	Review	Manual Therapy	2
2010	Ronan et al	Acupuncture and schizophrenia - Effect and acceptability: Preliminary results of the first UK study	Review	European Journal of Oriental Medicine	4
2010	Smith et al	Acupuncture for depression	Review	Cochrane Database of Systematic Reviews	1
2010	Son et al	Efficacy of ah shi point acupuncture on acne vulgaris	RCT	Acupuncture in Medicine	3
2010	Vickers et al	Individual patient data meta-analysis of acupuncture for chronic pain: Protocol of the Acupuncture Trialists' Collaboration	Protocol	Trials	1
2010	White et al	Does needling sensation (de qi) affect treatment outcome in pain? Analysis of data from a larger single-blind, randomised controlled trial	RCT	Acupuncture in Medicine	3
2010	Wu et al	Acupuncture in poststroke rehabilitation: A systematic review and meta-analysis of randomized trials	Review	Stoke	1

2010	Yeo et al	Consecutive acupuncture stimulations lead to significantly decreased neural responses	Observational study	Journal of Alternative and Complementary Medicine	3
2010	Zaringhalam et al	Reduction of chronic non-specific low back pain: A randomised controlled clinical trial on acupuncture and baclofen	RCT	Chinese Medicine	4
2010	Zaslawski	Ethical considerations for acupuncture and chinese herbal medicine clinical trials: A cross-cultural perspective	Commentary	Evidence-based Complementary and Alternative Medicine	4
2010	Zheng et al	Quality of randomized controlled trials in acupuncture treatment of hepatitis B virus infection - A systematic review	Review: research methodology discussions	Acupuncture and Electro-Therapeutics Research	4
2011	Asher et al	Quality of reporting on randomised controlled trials of auriculotherapy for pain	Review: research methodology discussions	Acupuncture in Medicine	3
2011	Bian et al	Consolidated standards of reporting trials (CONSORT) for traditional Chinese medicine: Current situation and future development	Review: research methodology discussions	Frontiers of Medicine in China	1
2011	Cohen et al	Acupuncture as analgesia for low back pain, ankle sprain and migraine in emergency departments: Study protocol for a randomized controlled trial	Protocol	Trials	1
2011	Cotchett et al	Effectiveness of trigger point dry needling for plantar heel pain: Study protocol for a randomised controlled trial	Protocol	Journal of Foot and Ankle Research	2
2011	Cotchett et al	Consensus for dry needling for plantar heel pain (plantar fasciitis): A modified Delphi study	Delphi study	Acupuncture in Medicine	3
2011	Hammerschlag et al	Randomized controlled trials of acupuncture (1997-2007): An assessment of reporting quality with a CONSORT- and STRICTA-based instrument	Review: research methodology discussions	Evidence-based Complementary and Alternative Medicine	4
2011	Kim et al	Acupuncture for symptom management in hemodialysis patients: A prospective, observational pilot study	Observational study	Journal of Alternative and Complementary Medicine	3
2011	Kim et al	The effectiveness of moxibustion: An overview during 10 Years	Review	Evidence-Based Complementary and Alternative Medicine	4

2011	Long et al	Exploring the evidence base for acupuncture in the treatment of Meniere's Syndrome-A systematic review	Review	Evidence-Based Complementary and Alternative Medicine	4
2011	Lu et al	Does acupuncture improve quality of life for patients with pain associated with the spine? A systematic review	Review	Evidence-Based Complementary and Alternative Medicine	4
2011	Man et al	Transcutaneous electrical nerve stimulation on ST36 and SP6 acupoints prevents hyperglycaemic response during anaesthesia: A randomised controlled trial	RCT	European Journal of Anaesthesiology	2
2011	Moher et al	Describing reporting guidelines for health research: A systematic review	Review: research methodology discussions	Journal of Clinical Epidemiology	2
2011	Molsberger et al	An international expert survey on acupuncture in randomized controlled trials for low back pain and a validation of the low back pain acupuncture score	Survey	European Journal of Medical Research	1
2011	Myung et al	Ameliorative effect of purple bamboo salt-pharmaceutical acupuncture on cisplatin-induced ototoxicity	Animal studies	Acta Oto-Laryngologica	2
2011	Paterson et al	Acupuncture for 'frequent attenders' with medically unexplained symptoms: A randomised controlled trial (CACTUS study)	RCT	British Journal of General Practice	1
2011	Price et al	Getting inside acupuncture trials - Exploring intervention theory and rationale	Review: research methodology discussions	BMC Complementary and Alternative Medicine	4
2011	Ronan et al	A case study exploration of the value of acupuncture as an adjunct treatment for patients diagnosed with schizophrenia: Results and future study design	Case series	Journal of Chinese Integrative Medicine	3
2011	Schmulson and Chang	Review article: The treatment of functional abdominal bloating and distension	Review	Alimentary Pharmacology and Therapeutics	1

2011	Schytt et al	Incompleteness of Swedish local clinical guidelines for acupuncture treatment during childbirth	Survey	Acta Obstetricia Et Gynecologica Scandinavica	2
2011	Smith et al	Development of an instrument to assess the quality of acupuncture: Results from a Delphi process	Guidelines related	Journal of Alternative and Complementary Medicine	3
2011	Stock-Schroer et al	Reporting experiments in homeopathic basic research-Description of the checklist development	Guidelines related	Evidence-Based Complementary and Alternative Medicine	4
2011	Stub et al	Acupuncture treatment for depression-A systematic review and meta-analysis	Review	European Journal of Integrative Medicine	4
2011	Wang and Young	Needling the pain and comforting the brain: Acupuncture in the treatment of chronic migraine	Editorial	Cephalalgia	2
2011	Wetzel et al	The effect of auricular acupuncture on fentanyl requirement during hip arthroplasty: A randomized controlled trial	RCT	Clinical Journal of Pain	2
2011	Wright and Aickin	Improvement of menopausal symptoms with acupuncture not reflected in changes to heart rate variability	Observational study	Acupuncture in Medicine	3
2011	Xiao et al	Assessing the quality of reports about randomized controlled trials of acupuncture treatment on mild cognitive impairment	Review: research methodology discussions	PLoS ONE	1
2011	Xu and Chen	Acupuncture: A paradigm of worldwide cross-cultural communication	Editorial	Chinese Journal of Integrative Medicine	4
2011	Yang et al	Acupuncture versus topiramate in chronic migraine prophylaxis: A randomized clinical trial	RCT	Cephalalgia	2
2011	Yeung et al	Electroacupuncture for residual insomnia associated with major depressive disorder: A randomized controlled trial	RCT	Sleep	2
2011	Yu et al	Effects of electroacupuncture on Benign Prostate Hyperplasia patients with lower urinary tract symptoms: A single-blinded, randomized controlled trial	RCT	Evidence-Based Complementary and Alternative Medicine	4

2011	Zhou et al	Acupuncture and auricular acupressure in relieving menopausal hot flashes of bilaterally ovariectomized Chinese women: A randomized controlled trial	RCT	Evidence-based Complementary and Alternative Medicine	4
2012	Chen et al	Designing and implementing multicenter clinical randomized controlled trials on moxibustion with large samples	Review: research methodology discussions	Journal of Traditional Chinese Medicine	4
2012	Chung et al	Randomized non-invasive sham-controlled pilot trial of electroacupuncture for postpartum depression	RCT	Journal of Affective Disorders	2
2012	Collazo Cha	Acupuncture and traditional Chinese dietary therapy in the treatment of patients with fibromyalgia. A randomized prospective study (Article in Spanish)	RCT	Revista Internacional de Acupuntura	4
2012	Duncan et al	Evaluating intense rehabilitative therapies with and without acupuncture for children with cerebral palsy: A randomized controlled trial	RCT	Archives of Physical Medicine and Rehabilitation	2
2012	Ferro et al	The combined effect of acupuncture and Tanacetum parthenium on quality of life in women with headache: Randomised study	RCT	Acupuncture in Medicine	3
2012	Iseri and Cabioglu	Migraine treatment and the role of acupuncture: A literature review	Review	Journal of the Australian Traditional-Medicine Society	4
2012	Lu et al	Acupuncture for dysphagia after chemoradiation in head and neck cancer: Rationale and design of a randomized, sham-controlled trial	Protocol	Contemporary Clinical Trials	1
2012	Manheimer et al	Acupuncture for treatment of irritable bowel syndrome	Review	Cochrane Database of Systematic Reviews	1
2012	Mathie et al	Method for appraising model validity of randomised controlled trials of homeopathic treatment: Multi-rater concordance study	Review: research methodology discussions	BMC Medical Research Methodology	1
2012	Patel et al	Massage for mechanical neck disorders	Review	Cochrane Database of Systematic Reviews	1

2012	Purepong et al	External validity in randomised controlled trials of acupuncture for osteoarthritis knee pain	Review: research methodology discussions	Acupuncture in Medicine	3
2012	Shuai et al	Issues of design and statistical analysis in controlled clinical acupuncture trials: An analysis of English-language reports from Western journals	Review: research methodology discussions	Statistics in Medicine	2
2012	Smith and Bauer	Traditional Chinese Medicine for cancer-related symptoms	Review	Seminars in Oncology Nursing	2
2012	Steinlechner	Be routinely recommended for the treatment of breech presentation?	Review	Journal of Chinese Medicine	4
2012	Tam et al	Citation classics in the integrative and complementary medicine literature: 50 frequently cited articles	Review	European Journal of Integrative Medicine	4
2012	van den Berg et al	Effect of acupuncture on pain and inflammation in patients with complex regional pain syndrome type 1. A small exploratory randomized controlled trial	RCT	Conference: 15th World Congress of Pain Clinicians	NA
2012	Xue et al	Effect of electroacupuncture on opioid consumption in patients with chronic musculoskeletal pain: protocol of a randomised controlled trial	Protocol	Trials	1
2012	Yang et al	Reports quality evaluation on acupuncture for treating acute attacks of migraine	Review: research methodology discussions	Chinese Journal of Evidence-Based Medicine	1
2012	Yao et al	Randomized controlled trial comparing acupuncture with placebo acupuncture for the treatment of Carpal Tunnel Syndrome	RCT	PM and R	1
2013	Bai and Lao	Neurobiological foundations of acupuncture: The relevance and future prospect based on neuroimaging evidence	Review	Evidence-based Complementary and Alternative Medicine	4
2013	Chen et al	Integrating acupuncture with exercise-based physical therapy for knee osteoarthritis: A randomized controlled trial	RCT	Journal of Clinical Rheumatology	2

2013	Collazo Chao and Muñoz Reina	Scalp acupuncture and acupunture for treatment of patients with fibromyalgia. Prospective randomized study (Article in Spanish)	RCT	Revista Internacional de Acupuntura	4
2013	Da Silva et al	Integrative medicine, integrative acupuncture	Editorial	European Journal of Integrative Medicine	4
2013	Deare et al	Acupuncture for treating fibromyalgia	Review	Cochrane Database of Systematic Reviews	1
2013	He et al	Acupuncture and moxibustion for cancer-related fatigue: A systematic review and meta-analysis	Review	Asian Pacific Journal of Cancer Prevention	2
2013	Kamioka et al	A checklist to assess the quality of reports on spa therapy and balneotherapy trials was developed using the Delphi consensus method: The SPAC checklist	Guidelines related	Complementary Therapies in Medicine	3 (2002)
2013	Li et al	Clinical effect of catgut implantation at acupoints for allergic rhinitis: study protocol for a randomized controlled trial	Protocol	Trials	1
2013	Lima et al	Acupuncture effectiveness as a complementary therapy in functional dyspepsia patients	RCT	Arquivos de Gastroenterologia	2
2013	Liu et al	Does traditional chinese medicine pattern affect acupoint specific effect? Analysis of data from a multicenter, randomized, controlled trial for primary dysmenorrhea	RCT	Journal of Alternative and Complementary Medicine	3
2013	Mannix et al	Acupuncture for managing phantom-limb syndrome: A systematic review	Review	Medical Acupuncture	3
2013	Manterola et al	Initiatives for reporting biomedical research results with different types of designs	Review: research methodology discussions	International Journal of Morphology	2
2013	McKeon et al	Acupuncture and acupressure for chemotherapy-induced nausea and vomiting: A systematic review	Review	Australian Journal of Acupuncture and Chinese Medicine	3
2013	Montgomery et al	The Oxford Implementation Index: A new tool for incorporating implementation data into systematic reviews and meta-analyses	Guidelines related	Journal of Clinical Epidemiology	2

2013	Paulson and Shay	Sympathetic nervous system responses to acupuncture and non-penetrating sham acupuncture in experimental forearm pain: A single-blind randomised descriptive study	RCT	Acupuncture in Medicine	3
2013	Shin et al	Effects of motion style acupuncture treatment in acute low back pain patients with severe disability: A multicenter, randomized, controlled, comparative effectiveness trial	RCT	Pain	2
2013	Sun et al	Electroacupuncture at Jing-jiaji points for neck pain caused by cervical spondylosis: A study protocol for a randomized controlled pilot trial	Protocol	Trials	1
2013	Yin et al	Therapeutic applications of herbal medicines for cancer patients	Review	Evidence-based Complementary and Alternative Medicine	4
2013	Zhang et al	Acupuncture as prophylaxis for menstrual-related migraine: Study protocol for a multicenter randomized controlled trial	Protocol	Trials	1
2013	Zhang et al	The preventive and therapeutic effect of acupuncture for radiation-induced xerostomia in patients with head and neck cancer: A systematic review	Review	Integrative Cancer Therapies	2
2014	Bryant et al	A systematic review of the quality of reporting in published smoking cessation trials for pregnant women: An explanation for the evidence-practice gap?	Review: research methodology discussions	Implementation Science	1
2014	Cayir et al	Acupuncture decreases matrix metalloproteinase-2 activity in patients with migraine	Observational study	Acupuncture in Medicine	3
2014	Chu et al	Reporting quality of English randomized controlled trials on acupuncture for neck disorders by the CONSORT statement and STRICTA	Review: research methodology discussions	Chinese Journal of Evidence-Based Medicine	1

2014	Collazo et al	Randomized prospective study to assess the effectiveness of several therapeutic procedures of traditional Chinese medicine in alleviation of pain and improvement in the standard of living in patients with fibromyalgia (Article in Spanish)	RCT	Revista Internacional de Acupuntura	4
2014	Gu et al	Assessment of registration information on methodological design of acupuncture RCTs: A review of 453 registration records retrieved from WHO International Clinical Trials Registry Platform	Review: research methodology discussions	Evidence-Based Complementary and Alternative Medicine	4
2014	Haddad and Palesh	Acupuncture in the treatment of cancer-related psychological symptoms	Review	Integrative Cancer Therapies	2
2014	Kamioka et al	Assessing the quality of study reports on spa therapy based on randomized controlled trials by the spa therapy checklist (SPAC)	Review: research methodology discussions	Complementary Therapies in Clinical Practice	4
2014	Kamioka et al	Effectiveness of animal-assisted therapy: A systematic review of randomized controlled trials	Review	Complementary Therapies in Medicine	3 (2002)
2014	Kamioka et al	Effectiveness of horticultural therapy: A systematic review of randomized controlled trials	Review	Complementary Therapies in Medicine	3 (2002)
2014	Lee et al	Effects of Wonli acupuncture procedure in patients with LSS: A clinical, retrospective study	Observational study	Evidence-Based Complementary and Alternative Medicine	4
2014	Luo et al	Quality of reporting of randomised controlled trials of acupuncture for neurological diseases conducted in China	Review: research methodology discussions	Acupuncture in Medicine	3
2014	Ortiz et al	A randomised multicentre trial of acupuncture in patients with seasonal allergic rhinitis - Trial intervention including physician and treatment characteristics	RCT related	BMC Complementary and Alternative Medicine	4
2014	Park et al	Acupuncture for the treatment of spasticity after stroke: A meta-analysis of randomized controlled trials	Review	Journal of Alternative and Complementary Medicine	3
2014	Stevens et al	Relation of completeness of reporting of health research to journals' endorsement of reporting guidelines: Systematic review	Review: research methodology discussions	BMJ	1

2014	Zhang et al	Electroacupuncture for pressure ulcer: A study protocol for a randomized controlled pilot trial	Protocol	Trials	1
From Chinese database (China National Knowledge Infrastructure)					
2003	Liu et al	Improving the quality of the report of acupuncture controlled trial by using CONSORT and STRICTA	Recommendations	Chinese Acupuncture and Moxibustion	4
2006	Zhang et al	Assessing the reporting quality of randomized controlled trials on acupuncture for Acute Ischemic Stroke using the CONSORT statement and STRICTA	Review: research methodology discussions	Chinese Journal of Evidence-Based Medicine	1
2007	Fei and Liu	Reporting interventions in clinical trials of acupuncture- The introduction and evaluation of the STRICTA standards	Recommendations	Journal of Traditional Chinese Medicine	4
2008	Zhu et al	Use of CONSORT and STRICTA to assess the quality of randomized controlled trials of acupuncture treatment of Functional Dyspepsia	Review: research methodology discussions	Shanghai Journal of Acupuncture and Moxibustion	4
2008	Lu et al	Assessing the reporting quality of randomized controlled trials on acupuncture for migraine using the CONSORT and STRICTA statement	Review: research methodology discussions	Journal of Chengdu University of Traditional Chinese Medicine	4
2008	Ai et al	Assessing the reporting quality of randomized controlled trials on acupuncture in children with Cerebral Palsy in China	Review: research methodology discussions	West China Medical Journal	1
2009	Liu	Clinical effect evaluation of a RCT for cotton-sheet moxibustion therapy in patients with Herpes Zoster	RCT	Doctoral dissertation in Chengdu University of Traditional Chinese Medicine	NA
2010	MacPherson et al	Revised standards for reporting interventions in clinical trials of acupuncture (STRICTA): Extending the consort statement (Chinese version)	Guidelines	Chinese Journal of Evidence-Based Medicine	1
2010	MacPherson et al	Revised STandards for Reporting Interventions in Clinical Trials of Acupuncture (STRICTA): Extending the CONSORT statement (Chinese version)	Guidelines	Journal of Chinese Integrative Medicine	3
2010	Sun et al	Assessment of the reporting quality of randomized controlled trials on acupuncture for simple obesity with the CONSORT statement and STRICTA	Review: research methodology discussions	Lishizhen Medicine and Materia Medica Research	4

2013	Zhao	Assessment of the reporting quality of randomized controlled trials on acupuncture for Perimenopausal Syndrome with the CONSORT statement and STRICTA	Review: research methodology discussions	Journal of Liaoning University of Traditional Chinese Medicine	4
2013	Chen	Assessment of the reporting quality of randomized controlled trials on acupuncture for Irritable Bowel Syndrome with the CONSORT statement and STRICTA	Review: research methodology discussions	Journal of Liaoning University of Traditional Chinese Medicine	4
2013	Zhong and Li	Evaluating the quality of clinical randomized controlled trials reports on acupuncture treating Chronic Atrophic Gastritis with CONSORT and STRICTA	Review: research methodology discussions	Lishizhen Medicine and Materia Medica Research	4
2013	Xu et al	Quality of reporting on randomised controlled trials of acupuncture for cervical vertigo	Review: research methodology discussions	Journal of Shaanxi College of Traditional Chinese Medicine	4
2013	Wang	Regulating-acupuncture for Allergic Rhinitis: A randomized controlled trial	RCT	Doctoral dissertation in Beijing University of Chinese Medicine	NA
2013	Sun	Quality of reporting on randomized controlled trials of acupuncture for Post-stroke Spastic Hemiplegic	Review: research methodology discussions	Master thesis in Beijing University of Chinese Medicine	NA
2014	Zhu et al	Reporting quality of English randomized controlled trials on acupuncture for Neck Disorders by the CONSORT statement and STRICTA	Review: research methodology discussions	Chinese Journal of Evidence-Based Medicine	1
2014	Wang et al	On quality of randomized controlled trial of Chronic Pelvic Inflammation treated with acupuncture and moxibustion	Review: research methodology discussions	Shandong Journal of Traditional Chinese Medicine	4
From Japanese database (Japan Science and Technology Information Aggregator, Electronic)					
2002	Tsukayama and Yamashta	Consensus recommendations for designing and reporting in clinical trials on acupuncture: An introduction of recommendations by STRICTA Group and IARF	Recommendations	Journal of the Japan Society of Acupuncture and Moxibustion	4
2005	Lee et al	Review of pragmatic clinical trials on acupuncture	Review	Journal of the Japan Society of Acupuncture and Moxibustion	4

2008	Kamioka et al	Significance of utilizing checklists and statements for improvement of the quality of studies concerning hot springs: Evidence grading and various study designs of epidemiological and clinical studies	Review: research methodology discussions	The Journal of the Japanese Society of Balneology, Climatology and Physical Medicine	4
2010	Nabeta et al	Practice and educational effects of a simulated randomized controlled trial for acupuncture school teachers	Observational study	Journal of the Japan Society of Acupuncture and Moxibustion	4
2013	MacPherson et al	Revised STandards for Reporting Interventions in Clinical Trials of Acupuncture (STRICTA): Extending the CONSORT statement (Japanese version)	Guidelines	Journal of the Japan Society of Acupuncture and Moxibustion	4
2014	Kamioka	A checklist to assess the quality of reports on spa therapy and balneotherapy trials: The SPAC checklist	Delphi study	The Journal of the Japanese Society of Balneology, Climatology and Physical Medicine	4
2014	Shimoichi et al	Acupuncture for patients with lower back pain-Systematic review of randomized controlled trials conducted in Japan	Reviews	Journal of the Japan Society of Acupuncture and Moxibustion	4

Journal types:
1. General medical non-CAM journals;
2. Specialty medical non-CAM journals;
3. CAM journals with STRICTA endorsement;
4. CAM journals without STRICTA endorsement.
