# Supplementary material for: STRICTA: is it time to do more?
Source: BMC Complement Altern Med. 2015 Jun 20;15:190. doi: 10.1186/s12906-015-0714-4 (PMC4474462; doi:10.1186/s12906-015-0714-4)
Supplement: Additional file 2: Table S2. — Characteristics of articles cited STRICTA 2010. The table presents the characteristics of the articles that cited the STRICTA guidelines 2010, including the title, author(s), publication year and journal, study type, and journal type, of the articles. [file 12906_2015_714_MOESM2_ESM.pdf]

Table S2. Characteristics of articles cited STRICTA 2010

Publication year	Author(s)	Article title	Study type	Journal name	Journal type
From English databases (Web of Science and Scopus)					
2010	Hopewell	Standards to improve the reporting of clinical trials in acupuncture	Commentary	Acupuncture in Medicine	3
2010	Kim et al	Psoas abscess caused by acupuncture?	Letter	Hemodialysis International	2
2010	MacPherson	Towards better reporting of interventions in clinical trials of acupuncture	Editorial	Journal of Chinese Integrative Medicine	3
2010	Moher	Reports of randomized trials: Ensure they are transparent, accurate, and complete	Editorial	Journal of Chinese Integrative Medicine	3
2010	O'Sullivan and Higginson	Clinical effectiveness and safety of acupuncture in the treatment of irradiation-induced xerostomia in patients with head and neck cancer: A systematic review	Review	Acupuncture in Medicine	3
2011	Asher et al	Quality of reporting on randomised controlled trials of auriculotherapy for pain	Review: research methodology discussions	Acupuncture in Medicine	3
2011	Bian et al	Consolidated standards of reporting trials (CONSORT) for traditional Chinese medicine: Current situation and future development	Review: research methodology discussions	Frontiers of Medicine in China	1
2011	Bian and Chang	Revised STRICTA as an extension of the CONSORT statement: More items should be involved in the checklist	Letter	Journal of Alternative and Complementary Medicine	3
2011	Burgos et al	Construction of a scale to assess methodological quality of diagnostic tests articles (Article in Spanish)	Guidelines related	Revista Chilena De Cirugia	2
2011	Corbett and Prestwich	A questionnaire survey to determine patient's knowledge, opinions and experience of acupuncture in an NHS GP practice	Letter	Acupuncture in Medicine	3

2011	Dhillon	Researching complementary and alternative therapies: Frameworks for evaluation	Review: research methodology discussions	Cancer Forum	2
2011	Enblom et al	The nonpenetrating telescopic sham needle may blind patients with different characteristics and experiences when treated by several therapists	RCT	Evidence-based Complementary and Alternative Medicine	4
2011	Enblom et al	Pilot testing of methods for evaluation of acupuncture for emesis during radiotherapy: A randomised single subject experimental design	RCT	Acupuncture in Medicine	3
2011	Eysenbach and Consort-Ehlth	CONSORT-EHEALTH: Improving and standardizing evaluation reports of web-based and mobile health interventions	Guidelines related	Journal of Medical Internet Research	1
2011	Hammerschlag et al	Randomized controlled trials of acupuncture (1997-2007): An assessment of reporting quality with a CONSORT- and STRICTA-based instrument	Review: research methodology discussions	Evidence-based Complementary and Alternative Medicine	4
2011	He et al	Quality assessment of reporting of randomization, allocation concealment, and blinding in traditional chinese medicine RCTs: A review of 3159 RCTs identified from 260 systematic reviews	Review: research methodology discussions	Trials	1
2011	Hopton and MacPherson	Assessing blinding in randomised controlled trials of acupuncture: Challenges and recommendations	Review: research methodology discussions	Chinese Journal of Integrative Medicine	4
2011	Jedel et al	Impact of electro-acupuncture and physical exercise on hyperandrogenism and oligo/amenorrhea in women with polycystic ovary syndrome: A randomized controlled trial	RCT	American Journal of Physiology - Endocrinology and Metabolism	2
2011	Johnson and Bjordal	Transcutaneous electrical nerve stimulation for the management of painful conditions: Focus on neuropathic pain	Review	Expert Review of Neurotherapeutics	2
2011	Kim et al	Acupuncture for premenstrual syndrome: A systematic review and meta-analysis of randomised controlled trials	Review	BJOG: An International Journal of Obstetrics and Gynaecology	2

2011	Kim et al	Moxibustion for managing type 2 diabetes mellitus: A systematic review	Review	Chinese Journal of Integrative Medicine	4
2011	Kim et al	RE: Hemopericardium following acupuncture?	Letter	Yonsei Medical Journal	1
2011	Knebel et al	Double-blinded, randomized controlled trial comparing real versus placebo acupuncture to improve tolerance of diagnostic esophagogastroduodenoscopy without sedation: A study protocol	Protocol	Trials	1
2011	Musil	The safety, acceptability, and effectiveness of acupuncture as an adjunctive treatment for acute symptoms in bipolar disorder (Article in German)	Commentary	Deutsche Zeitschrift fur Akupunktur	3
2011	Norrbrink and Lundeberg	Acupuncture and massage therapy for neuropathic pain following spinal cord injury: An exploratory study	Observational study	Acupuncture in Medicine	3
2011	Paley and Johnson	Acupuncture for cancer-induced bone pain: A pilot study	Observational study	Acupuncture in Medicine	3
2011	Pastore et al	True and sham acupuncture produced similar frequency of ovulation and improved LH to FSH ratios in women with polycystic ovary syndrome	RCT	Journal of Clinical Endocrinology and Metabolism	2
2011	Price et al	Getting inside acupuncture trials - Exploring intervention theory and rationale	Review: research methodology discussions	BMC Complementary and Alternative Medicine	4
2011	Raja-Khan et al	The physiological basis of complementary and alternative medicines for polycystic ovary syndrome	Review	American Journal of Physiology - Endocrinology and Metabolism	2
2011	Robinson et al	The evidence for Shiatsu: A systematic review of Shiatsu and acupressure	Review	BMC Complementary and Alternative Medicine	4
2011	Sarasà	Case report: Is acupuncture useful in the treatment of cluster headache? (Article in Spanish)	Case report	Revista Internacional de Acupuntura	4
2011	Skjeie et al	A pilot study of ST36 acupuncture for infantile colic	RCT	Acupuncture in Medicine	3
2011	Smith et al	Development of an instrument to assess the quality of acupuncture: Results from a Delphi process	Guidelines related	Journal of Alternative and Complementary Medicine	3

2011	Stener-Victorin and Manheimer	Commentary on the Cochrane review of acupuncture and assisted conception	Commentary	Explore: The Journal of Science and Healing	1
2011	Stub et al	Acupuncture treatment for depression-A systematic review and meta-analysis	Review	European Journal of Integrative Medicine	4
2011	Vas	How to write a case report: A guide for authors (Article in Spanish)	Guidelines	Revista Internacional de Acupuntura	4
2011	Witt	Clinical research on acupuncture - Concepts and guidance on efficacy and effectiveness research	Review: research methodology discussions	Chinese Journal of Integrative Medicine	4
2011	Wong et al	Acupuncture for acute management and rehabilitation of traumatic brain injury	Review	Cochrane Database of Systematic Reviews	1
2011	Xu and Chen	Acupuncture: A paradigm of worldwide cross-cultural communication	Editorial	Chinese Journal of Integrative Medicine	4
2012	Agbaje et al	Assessment of caries experience in epidemiological surveys: A review	Review: research methodology discussions	Community Dental Health	2
2012	Cao et al	An updated review of the efficacy of cupping therapy	Review	PLoS ONE	1
2012	Chen et al	Assessing the quality of reports about randomized controlled trials of acupuncture treatment on diabetic peripheral neuropathy	Review: research methodology discussions	PLoS ONE	1
2012	Collazo and Muñoz	Prediction of variability in the response to acupuncture in patients with chronic pain (Article in Spanish)	Observational study	Revista Internacional de Acupuntura	4
2012	Enblom et al	Acupuncture compared with placebo acupuncture in radiotherapy-induced nausea-A randomized controlled study	RCT	Annals of Oncology	2
2012	Förster and Stange	The CONSORT statement: The companion in pursuit of high quality phytotherapy research (Article in German)	Review: research methodology discussions	Zeitschrift fur Phytotherapie	2
2012	Fragoso and Ferreira	Immediate effects of acupuncture on biceps brachii muscle function in healthy and post-stroke subjects	RCT	Chinese Medicine	4

2012	Fragoso and Ferreira	Evaluation of the immediate effects of manual acupuncture on brachial bicep muscle function in healthy individuals and poststroke patients: A study protocol of a parallel-group randomized clinical trial	Protocol	Journal of Chinese Integrative Medicine	3
2012	Frisk et al	Acupuncture improves health-related quality-of-life (HRQoL) and sleep in women with breast cancer and hot flushes	RCT	Supportive Care in Cancer	2
2012	Fuller et al	Evaluating the impact and use of Transparent Reporting of Evaluations with Non-randomised Designs (TREND) reporting guidelines	Protocol for review	BMJ Open	1
2012	He et al	Target points: A discussion on acupuncture treatment of primary trigeminal neuralgia	Review	Journal of Chinese Integrative Medicine	3
2012	Hehir and Williams	A survey of health professionals' views about integration of Traditional Chinese Medicine (TCM) Acupuncture into NHS Scotland	Survey	European Journal of Integrative Medicine	4
2012	Hinman et al	Efficacy of acupuncture for chronic knee pain: protocol for a randomised controlled trial using a Zelen design	Protocol	BMC Complementary and Alternative Medicine	4
2012	Kim et al	Cupping for treating neck pain in video display terminal (VDT) users: A randomized controlled pilot trial	RCT	Journal of Occupational Health	2
2012	Kim et al	Intramuscular stimulation therapy for healthcare: A systematic review of randomised controlled trials	Review	Acupuncture in Medicine	3
2012	Lægaard	Akupunktur og forskning (Article in Danish)	Review	Ugeskrift for Laeger	1
2012	Lee et al	The effectiveness of acupuncture research across components of the trauma spectrum response (tsr): A systematic review of reviews	Review	Systematic Reviews	1
2012	Lee et al	Validation study of Kim's Sham needle by measuring facial temperature: An N-of-1 randomized double-blind placebo-controlled clinical trial	RCT	Evidence-based Complementary and Alternative Medicine	4

2012	Liang et al	The optimized acupuncture treatment for neck pain caused by cervical spondylosis: a study protocol of a multicentre randomized controlled trial	Protocol	Trials	1
2012	Lu et al	The feasibility and effects of acupuncture on quality of life scores during chemotherapy in ovarian cancer: Results from a pilot, randomized sham-controlled trial	RCT	Medical Acupuncture	3
2012	Lu et al	Acupuncture for dysphagia after chemoradiation in head and neck cancer: Rationale and design of a randomized, sham-controlled trial	Protocol	Contemporary Clinical Trials	1
2012	Lyons et al	Acupuncture and Chinese herbs as treatments for depression: An Australian pilot study	Observational study	Complementary Therapies in Clinical Practice	4
2012	MacPherson et al	Acupuncture for irritable bowel syndrome: Primary care based pragmatic randomised controlled trial	RCT	BMC Gastroenterology	2
2012	Manheimer et al	Acupuncture for treatment of irritable bowel syndrome	Review	Cochrane Database of Systematic Reviews	1
2012	Molassiotis et al	Acupuncture for cancer-related fatigue in patients with breast cancer: A pragmatic randomized controlled trial	RCT	Journal of Clinical Oncology	2
2012	Oravecz and Mészáros	Traditional Chinese medicine: Theoretical background and its use in China (Article in Hungarian)	Review	Orvosi Hetilap	1
2012	Pandolfi	The autumn of acupuncture	Review	European Journal of Internal Medicine	2
2012	Park et al	Effects of sweet bee venom pharmacopuncture treatment for chemotherapy-induced peripheral neuropathy: A case series	Case series	Integrative Cancer Therapies	2
2012	Purepong et al	External validity in randomised controlled trials of acupuncture for osteoarthritis knee pain	Review: research methodology discussions	Acupuncture in Medicine	3

2012	Qu et al	Does acupuncture improve the outcome of in vitro fertilization? Guidance for future trials	Review: research methodology discussions	European Journal of Integrative Medicine	4
2012	Serra	Good publication practice, its evolution and expected impact in public health	Review: research methodology discussions	Revista Cubana de Salud Publica	2
2012	Sherman	Guidelines for developing yoga interventions for randomized trials	Review: research methodology discussions	Evidence-based Complementary and Alternative Medicine	4
2012	Shin et al	Bee venom acupuncture for chronic low back pain: A randomised, sham-controlled, triple-blind clinical trial	RCT	European Journal of Integrative Medicine	4
2012	Smith et al	Using a Delphi consensus process to develop an acupuncture treatment protocol by consensus for women undergoing Assisted Reproductive Technology (ART) treatment	Delphi study	BMC Complementary and Alternative Medicine	4
2012	Stener-Victorin	Reply of the authors	Letter	Fertility and Sterility	2
2012	Stuardi and MacPherson	Acupuncture for irritable bowel syndrome: Diagnosis and treatment of patients in a pragmatic trial	RCT related	Journal of Alternative and Complementary Medicine	3
2012	Vixner et al	Manual and electroacupuncture for labour pain: Study design of a longitudinal randomized controlled trial	RCT related	Evidence-based Complementary and Alternative Medicine	4
2012	Wang et al	Scalp acupuncture for acute ischemic stroke: A meta-analysis of randomized controlled trials	Review	Evidence-based Complementary and Alternative Medicine	4
2012	Witt et al	Effectiveness guidance document (EGD) for acupuncture research - a consensus document for conducting trials	Guidelines	BMC Complementary and Alternative Medicine	4
2012	Witt et al	How well do randomized trials inform decision making: Systematic review using comparative effectiveness research measures on acupuncture for back pain	Review: research methodology discussions	PLoS ONE	1

2012	Wong et al	Acupuncture for acute management and rehabilitation of traumatic brain injury	Review	European Journal of Physical and Rehabilitation Medicine	2
2012	Tao et al	Randomized controlled trial comparing acupuncture with placebo acupuncture for the treatment of Carpal Tunnel Syndrome	RCT	PM and R	1
2012	Zaslawski and Garvey	Have you got consent? An overview of human research ethics for east asian medicine	Book chapter	Publisher: Nova Science Publishers, Inc.	NA
2012	Zhou et al	Acupuncture in treating hepatic fibrosis: A review with recommendation for future studies	Review	African Journal of Traditional Complementary and Alternative Medicines	4
2012	Zou et al	Traditional chinese herbal medicines for treating HIV infections and AIDS	Review	Evidence-based Complementary and Alternative Medicine	4
2013	Allam and Mohammed	The role of scalp acupuncture for relieving the chronic pain of degenerative osteoarthritis: A pilot study of egyptian women	Observational study	Medical Acupuncture	3
2013	Arvidsdotter et al	Effects of an integrative treatment, therapeutic acupuncture and conventional treatment in alleviating psychological distress in primary care patients - a pragmatic randomized controlled trial	RCT	BMC Complementary and Alternative Medicine	4
2013	Barbour t al	Better reporting of scientific studies: Why it matters	Editorial	PLoS Medicine	3
2013	Bosch et al	Sleep ameliorating effects of acupuncture in a psychiatric population	RCT	Evidence-based Complementary and Alternative Medicine	4
2013	Cao et al	Acupoint stimulation for fibromyalgia: A systematic review of randomized controlled trials	Review	Evidence-based Complementary and Alternative Medicine	4
2013	Chen et al	Manual acupuncture for treatment of diabetic peripheral neuropathy: A systematic review of randomized controlled trials	Review	PLoS ONE	1
2013	Chen et al	Effects of acupuncture treatment on depression insomnia: a study protocol of a multicenter randomized controlled trial	Protocol	Trial	1
2013	Cheng et al	Extending the CONSORT Statement to moxibustion	Guidelines	Journal of Chinese Integrative Medicine	3

2013	Cheong et al	The effectiveness of acupuncture in prevention and treatment of postoperative nausea and vomiting - A systematic review and meta-analysis	Review	PLoS ONE	1
2013	Cheong et al	Acupuncture and assisted reproductive technology	Review	Cochrane Database of Systematic Reviews	1
2013	Cho et al	Acupuncture for chronic low back pain: A multicenter, randomized, patient-assessor blind, sham-controlled clinical trial	RCT	Spine	2
2013	Da Silva et al	Integrative medicine, integrative acupuncture	Editorial	European Journal of Integrative Medicine	4
2013	D'Alessandro et al	Evaluation of acupuncture for Cancer symptoms in a Cancer Institute in Brazil	Observational study	Acupuncture in Medicine	3
2013	Deare	Reflections on undertaking the Cochrane review: 'Acupuncture for treating Fibromyalgia'	Commentary	Australian Journal of Acupuncture and Chinese Medicine	3
2013	Facco et al	Acupuncture versus valproic acid in the prophylaxis of migraine without aura: A prospective controlled study	RCT	Minerva Anestesiologica	2
2013	Ferreira et al	Laser acupuncture in patients with temporomandibular dysfunction: A randomized controlled trial	RCT	Lasers in Medical Science	2
2013	Gu et al	Registration quality assessment of acupuncture clinical trials	Review: research methodology discussions	PLoS ONE	1
2013	Heo et al	Acupuncture for spinal cord injury and its complications: A systematic review and meta-analysis of randomized controlled trials	Review	Evidence-based Complementary and Alternative Medicine	4
2013	Jiao et al	Review of Cochrane reviews on acupuncture: How Chinese resources contribute to Cochrane Reviews	Review: research methodology discussions	Journal of Alternative and Complementary Medicine	3
2013	Johansson et al	Acupuncture for ovulation induction in polycystic ovary syndrome: A randomized controlled trial	RCT	American Journal of Physiology - Endocrinology and Metabolism	2

2013	Kay Garcia et al	Systematic review of acupuncture in cancer care: A synthesis of the evidence	Review	Journal of Clinical Oncology	2
2013	Kim et al	Use of acupuncture for pain management in an academic Korean medicine hospital: A retrospective review of electronic medical records	Review	Acupuncture in Medicine	3
2013	Kim et al	The role of acupuncture in emergency department settings: A systematic review	Review	Complementary Therapies in Medicine	3 (2002)
2013	Kim et al	Acupuncture for posttraumatic stress disorder: A systematic review of randomized controlled trials and prospective clinical trials	Review	Evidence-based Complementary and Alternative Medicine	4
2013	Kinser and Robins	Control group design: Enhancing rigor in research of mind-body therapies for depression	Review: research methodology discussions	Evidence-based Complementary and Alternative Medicine	4
2013	Kuang et al	Acupuncture and clomiphene citrate for live birth in polycystic ovary syndrome: Study design of a randomized controlled trial	Protocol	Evidence-based Complementary and Alternative Medicine	4
2013	Landgren	Acupuncture in practice: Investigating acupuncturists' approach to treating infantile colic	Survey	Evidence-based Complementary and Alternative Medicine	4
2013	Lee et al	Scalp acupuncture for Parkinson’s disease: A systematic review of randomized controlled trials	Review	Chinese Journal of Integrative Medicine	4
2013	Lee et al	Acupuncture for acute low back pain: A systematic review	Review	Clinical Journal of Pain	2
2013	Lee et al	Moxibustion for treating knee osteoarthritis: Study protocol of a multicentre randomised controlled trial	Protocol	BMC Complementary and Alternative Medicine	4
2013	Lee et al	Scalp acupuncture for stroke recovery: A systematic review and meta-analysis of randomized controlled trials	Review	European Journal of Integrative Medicine	4
2013	Lee et al	Acupuncture for gouty arthritis: A concise report of a systematic and meta-analysis approach	Review	Rheumatology	2
2013	Liang et al	Deqi sensation in placebo acupuncture: A crossover study on chinese medicine students	RCT	Evidence-based Complementary and Alternative Medicine	4

2013	Lu et al	Quality of reporting and its correlates among randomized controlled trials on acupuncture for cancer pain: Application of the CONSORT 2010 Statement and STRICTA	Review: research methodology discussions	Expert Review of Anticancer Therapy	2
2013	Macpherson et al	Acupuncture for depression: Patterns of diagnosis and treatment within a randomised controlled trial	RCT related	Evidence-based Complementary and Alternative Medicine	4
2013	Marx et al	AcuTrials®: An online database of randomized controlled trials and systematic reviews of acupuncture	Recommendations	BMC Complementary and Alternative Medicine	4
2013	McLay et al	Research methodology for the study of complementary and alternative medicine in the treatment of military PTSD	Review: research methodology discussions	Psychiatric Annals	2
2013	Molassiotis et al	A randomized, controlled trial of acupuncture self-needling as maintenance therapy for cancer-related fatigue after therapist-delivered acupuncture	RCT	Annals of Oncology	2
2013	Nedeljković et al	Modalities of acupuncture treatments in assisted reproductive technology - A comparison of treatment practice in Swiss, German, and Austrian fertility centers with findings from randomized controlled trials (Article in German)	Survey	Forschende Komplementarmedizin	4
2013	Oh et al	Acupuncture for treatment of arthralgia secondary to aromatase inhibitor therapy in women with early breast cancer: Pilot study	RCT	Acupuncture in Medicine	3
2013	O'Leary and Crawford	Review article: Reporting guidelines in the biomedical literature	Review: research methodology discussions	Canadian Journal of Anesthesia	2
2013	Park et al	Acupuncture for ankle sprain: Systematic review and meta-analysis	Review	BMC Complementary and Alternative Medicine	4
2013	Park et al	Moxibustion in the management of irritable bowel syndrome: Systematic review and meta-analysis	Review	BMC Complementary and Alternative Medicine	4

2013	Rashidi et al	Effects of acupuncture on the outcome of in vitro fertilisation and intracytoplasmic sperm injection in women with polycystic ovarian syndrome	RCT	Acupuncture in Medicine	3
2013	Rivers and Zollman	Reliability of surface acupuncture point location	Observational study	Medical Acupuncture	3
2013	Sardella et al	Acupuncture and burning mouth syndrome: A pilot study	Observational study	Pain Practice	2
2013	Simcock et al	Arix: A randomised trial of acupuncture V oral care sessions in patients with chronic xerostomia following treatment of head and neck cancer	RCT	Annals of Oncology	2
2013	Skjeie and Gardasevic	Medical acupuncture modality: Principles, explanatory model, and scientific developments during 2005-2012	Review	Journal of Acupuncture and Tuina Science	4
2013	Smith et al	The effect of acupuncture on post-cancer fatigue and well-being for women recovering from breast cancer: A pilot randomised controlled trial	RCT	Acupuncture in Medicine	3
2013	Towler et al	What is the evidence for the use of acupuncture as an intervention for symptom management in cancer supportive and palliative care: An integrative overview of reviews	Review	Supportive Care in Cancer	2
2013	Turner et al	An evaluation of epidemiological and reporting characteristics of Complementary and Alternative Medicine (CAM) systematic reviews (SRs)	Review: research methodology discussions	PLoS ONE	1
2013	Wong et al	Acupuncture for acute management and rehabilitation of traumatic brain injury	Review	Cochrane Database of Systematic Reviews	1
2013	Xu et al	Acupuncture for chronic low back pain in long-term follow-up: A meta-analysis of 13 randomized controlled trials	Review	American Journal of Chinese Medicine	4
2013	Xu et al	Effect of acupuncture treatment for weight loss on gut flora in patients with simple obesity	Letter	Acupuncture in Medicine	3
2013	Yang et al	Meta-analysis of acupuncture for relieving non-organic dyspeptic symptoms suggestive of diabetic gastroparesis	Review	BMC Complementary and Alternative Medicine	4

2013	Yang et al	Factors contributing to de Qi in acupuncture randomized clinical trials	Review	Evidence-based Complementary and Alternative Medicine	4
2013	Yun et al	Effect of facial cosmetic acupuncture on facial elasticity: An open-label, single-arm pilot study	Observational study	Evidence-based Complementary and Alternative Medicine	4
2013	Zhang et al	Ear acupressure for smoking cessation: Study protocol for a randomised controlled trial	Protocol	Forschende Komplementarmedizin	4
2013	Zhang et al	Clinical research of traditional Chinese medicine needs to develop its own system of core outcome sets	Review: research methodology discussions	Evidence-based Complementary and Alternative Medicine	4
2014	Abe et al	Acupuncture for treating persistent pain in Brazilian para-athletes	Observational study	Medical Acupuncture	3
2014	Alraek	Designing clinical studies that take into account traditional East Asian medicine's systems and methods - With focus on pattern identification	Review: research methodology discussions	Chinese Journal of Integrative Medicine	4
2014	Alraek	Just to be sure	Letter	Acupuncture in Medicine	3
2014	Anderson et al	Faculty survey to assess research literacy and evidence-informed practice interest and support at Pacific College of Oriental Medicine	Survey	Journal of Alternative and Complementary Medicine	3
2014	Appleyard et al	Should systematic reviews assess the risk of bias from sham-placebo acupuncture control procedures?	Review: research methodology discussions	European Journal of Integrative Medicine	4
2014	Bae et al	Efficacy of acupuncture in reducing preoperative anxiety: A meta-analysis	Review	Evidence-based Complementary and Alternative Medicine	4
2014	Baeumler et al	Effects of acupuncture on sensory perception: A systematic review and meta-analysis	Review	PLoS ONE	1
2014	Cho et al	Acupuncture with non-steroidal anti-inflammatory drugs (NSAIDs) versus acupuncture or NSAIDs alone for the treatment of chronic neck pain: An assessor-blinded randomised controlled pilot study	RCT	Acupuncture in Medicine	3
2014	Choi et al	Acupuncture for the treatment of chronic obstructive pulmonary disease: A protocol of a systematic review	Protocol for review	BMJ Open	1

2014	Choi et al	Endorsement for improving the quality of reports on randomized controlled trials of traditional medicine journals in Korea: A systematic review	Review: research methodology discussions	Trials	1
2014	Cooke et al	PC6 acupoint stimulation for the prevention of postcardiac surgery nausea and vomiting: A protocol for a two-group, parallel, superiority randomised clinical trial	Protocol	BMJ Open	1
2014	Crawford et al	The current state of the science for active self-care Complementary and Integrative Medicine therapies in the management of chronic pain symptoms: Lessons learned, directions for the future	Review: research methodology discussions	Pain Medicine	2
2014	Gadau et al	Acupuncture and moxibustion for lateral elbow pain: A systematic review of randomized controlled trials	Review	BMC Complementary and Alternative Medicine	4
2014	Garrow et al	Role of acupuncture in the management of diabetic painful neuropathy (DPN): A pilot RCT	RCT	Acupuncture in Medicine	3
2014	Glickman-Simon and Lepper	Panax Ginseng for psychomotor performance and cognition, Spinal manipulation for Lumbar Disk Herniation, Ginger for Migraines, Music therapy for mental illness, and acupuncture for Diabetic Peripheral Neuropathy	General article: expert opinion	Explore- The Journal of Science and Healing	4
2014	Haddad and Palesh	Acupuncture in the treatment of cancer-related psychological symptoms	Review	Integrative Cancer Therapies	2
2014	Hadianfard et al	Efficacy of acupuncture versus local Methylprednisolone Acetate injection in De Quervain's Tenosynovitis: A randomized controlled trial	RCT	Journal of Acupuncture and Meridian Studies	4
2014	Hammerschlag et al	Nontouch biofield therapy: A systematic review of human randomized controlled trials reporting use of only nonphysical contact treatment	Review	Journal of Alternative and Complementary Medicine	3

2014	Han et al	Acupuncture for functional dyspepsia: Study protocol for a two-center, randomized controlled trial	Protocol	Trials	1
2014	Han et al	Effect of acupuncture on patients with insomnia: Study protocol for a randomized controlled trial	Protocol	Trials	1
2014	Hasegawa et al	Acupuncture for acute non-specific low back pain: a randomised, controlled, double-blind, placebo trial	RCT	Acupuncture in Medicine	3
2014	Hinman et al	Acupuncture for chronic knee pain: A randomized clinical trial	RCT	Journal of the American Medical Association	1
2014	Hoffmann et al	Better reporting of interventions: Template for intervention description and replication (TIDieR) checklist and guide	Guidelines	BMJ (Online)	1
2014	Im et al	Effects of acupuncture at GB20 on CO_2 reactivity in the Basilar and Middle Cerebral Arteries during Hypocapnia in healthy participants	Observational study	Journal of Alternative and Complementary Medicine	3
2014	Jerng et al	The effectiveness and safety of acupuncture for poor semen quality in infertile males: A systematic review and meta-analysis	Review	Asian Journal of Andrology	2
2014	Kearney	Hoping for a TREND toward PRISMA: The variety and value of research reporting guidelines	Editorial	Research in Nursing and Health	2
2014	Kim et al	Acupuncture as analgesia for non-emergent acute non-specific neck pain, ankle sprain and primary headache in an emergency department setting: A protocol for a parallel group, randomised, controlled pilot trial	Protocol	BMJ Open	1
2014	Kim et al	Assessment of the quality of reporting for treatment components in Cochrane reviews of acupuncture	Review: research methodology discussions	BMJ Open	1
2014	Kim et al	Assessment of the quality of reporting in randomised controlled trials of acupuncture in the Korean literature using the CONSORT statement and STRICTA guidelines	Review: research methodology discussions	BMJ Open	1

2014	Kim and Kang	Rebuttal to "Life-threatening cardiac tamponade: a rare complication of acupuncture": Who framed acupuncture?	Letter	Journal of Cardiothoracic Surgery	2
2014	Lee et al	Acupuncture for postoperative pain in laparoscopic surgery: A systematic review protocol	Protocol for review	BMJ Open	1
2014	Legro et al	Improving the reporting of clinical trials of Infertility treatment (IMPRINT): Explanation and elaboration of the modification of the CONSORT statement	Guidelines	Fertility and Sterility	2
2014	Legro et al	Improving the reporting of clinical trials of Infertility treatments (IMPRINT): Modifying the CONSORT statement	Guidelines related	Fertility and Sterility	2
2014	Legro et al	Improving the reporting of clinical trials of Infertility treatments (IMPRINT): Modifying the CONSORT statement	Guidelines related	Human Reproduction	2
2014	Luo et al	Quality of reporting of randomised controlled trials of acupuncture for neurological diseases conducted in China	Review: research methodology discussions	Acupuncture in Medicine	3
2014	Mandiroglu et al	Acupuncture for neuropathic pain due to bortezomib in a patient with multiple myeloma	Case report	Acupuncture in Medicine	3
2014	Martins et al	Factors influencing further acupuncture usage and a more positive outcome in patients with Osteoarthritis of the knee and the hip: A 3-year follow-up of a randomized pragmatic trial	RCT	Clinical Journal of Pain	2
2014	Miller et al	Benefits of acupuncture for diabetic gastroparesis: a comparative preliminary study	Observational study	Acupuncture in Medicine	3
2014	Moon et al	Acupuncture for treating whiplash associated disorder: A systematic review of randomised clinical trials	Review	Evidence-based Complementary and Alternative Medicine	4
2014	Moreno-Martín and Santana-Pineda	Is acupuncture effective in treatment for cervicobrachial chronic pain? Qualitative analysis of the literature (Article in Spanish)	Review	Revista Internacional de Acupuntura	4

2014	Nandi et al	Acupuncture in IVF: A review of current literature	Review	Journal of Obstetrics and Gynaecology	2
2014	Ong and Claydon	The effect of dry needling for myofascial trigger points in the neck and shoulders: A systematic review and meta-analysis	Review	Journal of Bodywork and Movement Therapies	1
2014	Park et al	The safety of acupuncture during pregnancy: A systematic review	Review	Acupuncture in Medicine	3
2014	Phillips et al	A systematic review of how studies describe educational interventions for evidence-based practice: Stage 1 of the development of a reporting guideline	Review: research methodology discussions	BMC Medical Education	2
2014	Pirotta et al	Acupuncture for menopausal vasomotor symptoms: Study protocol for a randomised controlled trial	Protocol	Trials	1
2014	Seo et al	Bee venom acupuncture, NSAIDs or combined treatment for chronic neck pain: Study protocol for a randomized, assessor-blind trial	Protocol	Trials	1
2014	Stevens et al	Relation of completeness of reporting of health research to journals' endorsement of reporting guidelines: Systematic review	Review: research methodology discussions	BMJ	1
2014	Vixner et al	Acupuncture with manual and electrical stimulation for labour pain: A longitudinal randomised controlled trial	RCT	BMC Complementary and Alternative Medicine	4
2014	Wang et al	Comparison of clinical effectiveness of acupuncture and a western drug on allergic rhinitis: Study protocol for a randomized controlled trial	Protocol	Journal of Traditional Chinese Medicine	4
2014	Ward et al	Components and reporting of yoga interventions for musculoskeletal conditions: A systematic review of randomised controlled trials	Review: research methodology discussions	Complementary Therapies in Medicine	3 (2002)
2014	Witt et al	Effectiveness guidance document (EGD) for Chinese medicine trials: A consensus document	Guidelines	Trials	1

2014	Yeo et al	Acupuncture on GB34 activates the precentral gyrus and prefrontal cortex in Parkinson's disease	Observational study	BMC Complementary and Alternative Medicine	4
2014	Yeo et al	Randomised clinical trial of five ear acupuncture points for the treatment of overweight people	RCT	Acupuncture in Medicine	3
2014	Young	Acupuncture and moxibustion may increase pregnancy rates in IVF after embryo implantation failure	General article: expert opinion	Integrative Medicine Alert	4
2014	Yu et al	Quality assessment of randomized controlled trials reporting on knee osteoarthritis treated with warming needle moxibustion	Review: research methodology discussions	Journal of Traditional Chinese Medicine	4
2014	Zhang et al	Sham control methods used in ear-acupuncture/ear-acupressure randomized controlled trials: A systematic review	Review	Journal of Alternative and Complementary Medicine	3
2014	Zhuang et al	Quality of reporting on randomized controlled trials of acupuncture for stroke rehabilitation	Review: research methodology discussions	BMC Complementary and Alternative Medicine	4
2014	Zorzela et al	Quality of reporting in systematic reviews of adverse events: Systematic review	Review: research methodology discussions	BMJ (Online)	1
From Chinese database (China National Knowledge Infrastructure)					
2011	Tang et al	Acupuncture and guasha for stiff neck: A randomized controlled trial	RCT	Jilin Journal of Traditional Chinese Medicine	4
2011	Fan	The literature evaluation and clinical research of treating depression with acupuncture and moxibustion	RCT, Review	Doctoral dissertation in Guangzhou University of Chinese Medicine	NA
2011	Qu	The evaluating quality research of randomized controlled trials literature on acupuncture and moxibustion on treating Scapulohumeral Periarthritis	Review: research methodology discussions	Master thesis in China Academy of Chinese Medical Sciences	NA
2012	Lin and Wu	Deconstruction of the clinical research model for acupuncture effects	General article: expert opinion	Chinese Acupuncture and Moxibustion	4

2012	He et al	Target points: A discussion on acupuncture treatment of primary trigeminal neuralgia	General article: expert opinion	Journal of Chinese Integrative Medicine	3
2012	Wang and Liang	Thoughts and prospects of research on acupoints compatibility	General article: expert opinion	Chinese Acupuncture and Moxibustion	4
2012	Zhang et al	Assessing the reporting quality of randomized controlled trials on acupuncture for Diabetic Gastroparesis using the CONSORT statement and STRICTA	Review: research methodology discussions	Tianjin Journal of Traditional Chinese Medicine	4
2012	Zhang et al	Methodological quality of randomized controlled trials of acupuncture for sudden hearing loss	Review: research methodology discussions	Shanghai Journal of Acupuncture and Moxibustion	4
2012	Liu	Research on the quality of systematic reviews and randomized controlled trials of acupuncture and congnition of reporting guideline	Review: research methodology discussions	Doctoral dissertation in Lanzhou University	NA
2012	Liu	Lower extremity point acupuncture treatment of periarthritis of shoulder with shoulder movement clinical curative effect observation	RCT	Master thesis in Heilongjiang University of Chinese Medicine	NA
2012	Shi	An evaluation research of literature and clinical trial with the Back-Shu and Front-Mu acupoints of Stomach for treating Diabetic Gastroparesis	RCT, Review: research methodology discussions	Master thesis in Chengdu University of Traditional Chinese Medicine	NA
2012	Zhang	Research of clinic guide of Chinese Medicine for Nutritional Iron Deficiency Anemia	Guidelines related	Master thesis in Nanjing University of Chinese Medicine	NA
2013	Liu et al	Quality improvement on acupuncture intervention report: Application and perfetion of STRICTA	Commentary	Chinese Acupuncture and Moxibustion	4
2013	Qu et al	Quality of reporting on randomized controlled trials of acupuncture for Scapulohumeral Periarthritis: A survey	Survey	Chinese Journal of Basic Medicine in Traditional Chinese Medicine	4
2013	Shen et al	Quality evaluation of the randomized controlled trials on acupuncture-moxibustion for Primary Trigeminal Neuralgia by using CONSORT and STRICTA	Review: research methodology discussions	Shanghai Journal of Acupuncture and Moxibustion	4

2013	Wang and Zhao	Quality of research report in China according to the STRICTA of 2010 edition-Examples of RCT reports from acupuncture trials for insomnia in recent two years	Review: research methodology discussions	Journal of Clinical Acupuncture and Moxibustion	4
2013	Xu et al	Assessment of reporting quality of randomized controlled trials on acupuncture for Shoulder-hand Syndrome with CONSORT statement and STRICTA	Review: research methodology discussions	Liaoning Journal of Traditional Chinese Medicine	4
2013	Zhang	Clinical study on the Postherpetic Neuralgia with round acupuncture and its effect on IL-6	RCT	Master thesis in Nanjing University of Chinese Medicine	NA
2013	Zhang	A systematic review and the GRADE of acupuncture and moxibustion treatment for sudden hearing loss	Review	Master thesis in Nanjing University of Chinese Medicine	NA
2014	Jiang et al	Assessment of reporting quality of randomized controlled trials on acupuncture for Leukocytopenia with CONSORT statement and STRICTA	Review: research methodology discussions	Modern Journal of Integrated Traditional Chinese and Western Medicine	4
2014	Liu et al	Study on the normative statements elements of randomized controlled trial literature on ulcerative colitis treated by acupuncture and moxibustion	Review: research methodology discussions	China Journal of Traditional Chinese Medicine and Pharmacy	4
From Japanese database (Japan Science and Technology Information Aggregator, Electronic)					
2012	Kim et al	Cupping for treating neck pain in video display terminal (VDT) users: A randomized controlled pilot trial	RCT	Journal of Occupational Health	2

Journal types:
1. General medical non-CAM journals;
2. Specialty medical non-CAM journals;
3. CAM journals with STRICTA endorsement;
4. CAM journals without STRICTA endorsement.
